# Pan-sarbecovirus prophylaxis with human anti-ACE2 monoclonal antibodies

**DOI:** 10.1038/s41564-023-01389-9

**Published:** 2023-05-15

**Authors:** Fengwen Zhang, Jesse Jenkins, Renan V. H. de Carvalho, Sandra Nakandakari-Higa, Teresia Chen, Morgan E. Abernathy, Viren A. Baharani, Elisabeth K. Nyakatura, David Andrew, Irina V. Lebedeva, Ivo C. Lorenz, H.-Heinrich Hoffmann, Charles M. Rice, Gabriel D. Victora, Christopher O. Barnes, Theodora Hatziioannou, Paul D. Bieniasz

**Affiliations:** 1 Laboratory of Retrovirology, The Rockefeller University, New York, NY USA; 2 Laboratory of Lymphocyte Dynamics, The Rockefeller University, New York, NY USA; 3 Department of Biology, Stanford University, Stanford, CA USA; 4 Tri-Institutional Therapeutics Discovery Institute, New York, NY USA; 5 Laboratory of Virology and Infectious Disease, The Rockefeller University, New York, NY USA; 6 Chan Zuckerberg Biohub, San Francisco, CA USA; 7 Howard Hughes Medical Institute, The Rockefeller University, New York, NY USA

**Keywords:** SARS-CoV-2, Viral infection

## Abstract

Human monoclonal antibodies (mAbs) that target the severe acute respiratory syndrome coronavirus 2 (SARS-CoV-2) spike protein have been isolated from convalescent individuals and developed into therapeutics for SARS-CoV-2 infection. However, therapeutic mAbs for SARS-CoV-2 have been rendered obsolete by the emergence of mAb-resistant virus variants. Here we report the generation of a set of six human mAbs that bind the human angiotensin-converting enzyme-2 (hACE2) receptor, rather than the SARS-CoV-2 spike protein. We show that these antibodies block infection by all hACE2 binding sarbecoviruses tested, including SARS-CoV-2 ancestral, Delta and Omicron variants at concentrations of ~7–100 ng ml^−1^. These antibodies target an hACE2 epitope that binds to the SARS-CoV-2 spike, but they do not inhibit hACE2 enzymatic activity nor do they induce cell-surface depletion of hACE2. They have favourable pharmacology, protect hACE2 knock-in mice against SARS-CoV-2 infection and should present a high genetic barrier to the acquisition of resistance. These antibodies should be useful prophylactic and treatment agents against any current or future SARS-CoV-2 variants and might be useful to treat infection with any hACE2-binding sarbecoviruses that emerge in the future.

## Abstract (web summary)

A suite of human monoclonal antibodies block infection by all human ACE2 binding sarbecoviruses, including severe acute respiratory syndrome coronavirus 2 variants, at subnanomolar concentrations in cell culture and protect mice against severe acute respiratory syndrome coronavirus 2 infection.

## Main

Human monoclonal antibodies (mAbs) can be used therapeutically to confer a state of passive immunity^1,2^ and have been developed to target the severe acute respiratory syndrome coronavirus 2 (SARS-CoV-2) spike protein as both therapeutic and preventive agents^2–7^. However, therapeutic or prophylactic use of spike-targeting antibodies against SARS-CoV-2 in particular, and viruses in general, has two main drawbacks.

First, mAbs developed for therapy are produced by human immune systems, and the most potent of them are very similar to neutralizing antibodies commonly elicited by infection or vaccination^2,8,9^. Over time, as SARS-CoV-2 has replicated in human populations that have immunity owing to either infection, vaccination or both, the virus has frequently encountered naturally elicited neutralizing antibodies and has thus evolved to become resistant to therapeutic mAbs, even when they have not been widely used^10–13^. Indeed, emergent SARS-CoV-2 variants have rendered obsolete most SARS-CoV-2 mAbs that were generated from immune repertoires of individuals exposed to SARS-CoV-2 or its emergent variants^13,14^.

Second, while it is might seem prudent to stockpile sarbecovirus spike-targeting mAbs in anticipation of future pandemics, forecasting which virus species might emerge or cause disease is difficult. For example, SARS-CoV-2 is one of three recently emergent coronaviruses that are antigenically distinct from each other^15^. It is therefore not feasible to pre-emptively generate spike-targeting mAb therapeutics or prophylactics that offer reliable and effective protection against an emergent virus. Ideally, mAbs developed in anticipation of future emergent viral disease would be resilient to mutations that arise during epidemic spread and effective against entire classes of viruses.

Sarbecoviruses, including SARS-CoV, SARS-CoV-2 and SARS-related coronaviruses in bats and other mammals, use angiotensin-converting enzyme-2 (ACE2) as their primary functional receptor^16,17^. In principle, antivirals against sarbecoviruses could target human (h)ACE2 rather than spike proteins. Resistance would require a profound change in how sarbecoviruses interact with hACE2, or acquisition of the ability to use a new receptor, both of which are likely to be high genetic hurdles. It is possible that mAbs targeting a self-molecule (such as ACE2) might cause side effects, but there are precedents for using mAbs or small molecules to block receptors, particularly in situations when the viral or cellular ligands are too variable to be blocked by single mAbs. For example, the HIV-1 receptor (CD4)-binding antibody ibalizumab is approved to treat patients infected with multidrug-resistant HIV-1 (ref. ^18^). Likewise, a CCR5-binding mAb, leronlimab, protects monkeys against mucosal simian/human immunodeficiency virus (SHIV) transmission^19^. A small molecule targeting CCR5, maraviroc, has been developed as an anti-HIV-1 therapeutic^20^. Therapeutic receptor-blocking antibodies include those used in interferonopathies; the 16 type-I interferons are too variable to be neutralized by a single mAb, but anifrolumab, a mAb that ablates interferon action by binding the type-I IFN receptor, is used as a treatment for systemic lupus erythematosus^21,22^. Crucially, self-targeting mAbs can be made safe for use in humans by engineering Fc domains to ablate cytotoxic effector functions^23–25^.

In this Article, we produced a suite of human mAbs that bind hACE2 with affinities in the low nanomolar to picomolar range. These mAbs block infection by pseudotypes of all tested sarbecoviruses, with potencies that approach those of SARS-CoV-2 spike targeting therapeutic mAbs. A 3.3 Å cryo-electron microscopy (cryo-EM) structure of one such mAb bound to hACE2 shows recognition of the α1 helix and competition with the spike receptor binding domain (RBD). The anti-hACE2 mAbs do not inhibit hACE2 enzymatic activity or induce hACE2 internalization and, importantly, have favourable pharmacology and provide protection against SARS-CoV-2 lung infection in hACE2 knock-in mice. The mAbs described herein may thus be used as prophylactic and treatment agents against any emergent SARS-CoV-2 variant and future sarbecovirus pandemic threats.

## Results

### Production of hACE2-binding human mAbs

**Fig. 1:**
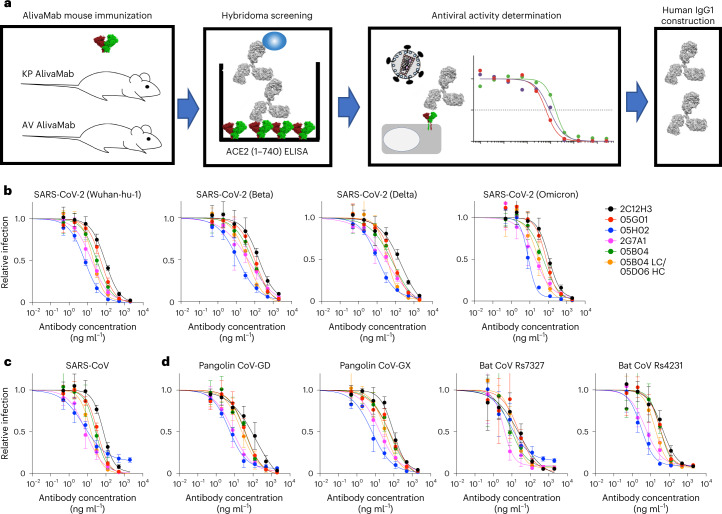
Human anti-hACE2 antibodies that block sarbecovirus pseudotype infection. **a**, Recombinant hACE2 extracellular domain (1–740aa) as His-tagged (monomer) or Fc-fused (dimer) proteins were injected into KP AlivaMab mice that generate human Kappa (κ) light chains or AV AlivaMab that generate both human Kappa (κ) and human Lambda (λ) light chains. ELISA screening yielded 82 reactive hybridoma clones. The supernatants of positive hybridoma clones were tested for inhibition of SARS-CoV-2 spike-pseudotyped virus infection, and thereafter ten human IgG1 antibodies were constructed. **b**–**d**, Inhibition of HIV-1-based pseudotyped virus infection by anti-hACE2 mAbs. Six fully human anti-hACE2 antibodies (2C12H3, 05G01, 05H02, 2G7A1, 05B04 and the hybrid antibody 05B04LC/05D06HC) were incubated with Huh-7.5 target cells, and cells were infected with pseudotypes bearing spike proteins from SARS-CoV-2 variants (**b**), SARS-CoV (**c**) or pangolin and bat sarbecoviruses (**d**), with the sarbecovirus spike proteins indicated. Infection was measured using NanoLuc luciferase assays. Mean and range of four independent experiments is plotted. Source data

**Extended Data Fig. 1:**
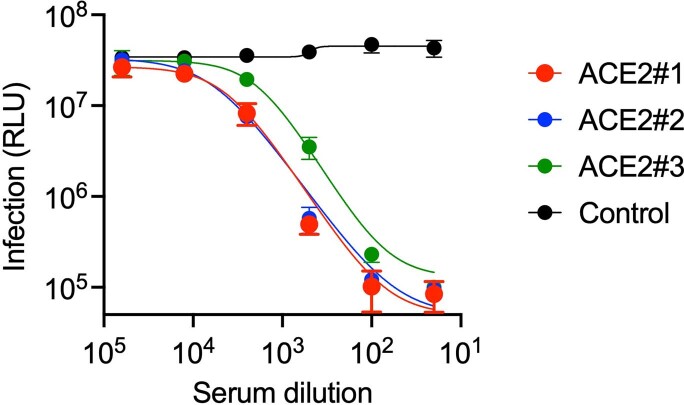
Inhibition of HIV-1 based pseudotyped virus infection by anti-hACE2 mAbs mouse serum. Heat inactivated serum from hACE2-8XHis immunized mice or an unimmunized control was incubated with Huh-7.5 target cells. Cells were then infected with SARS-CoV-2 pseudotyped HIV-1 and infection was measured using NanoLuc luciferase assay 48 h later. Mean and range of four independent experiments is plotted. Source data

In a pilot experiment, BALB/c6 mice were immunized with recombinant hACE2 extracellular domain (1–740aa). Sera collected at 35 days after immunization showed potent antiviral activity against SARS-CoV-2 pseudotyped viruses, with 50% inhibitory titres in the range 1,860–6,050 (Extended Data Fig. 1), consistent with findings that murine mAbs targeting hACE2 can inhibit sarbecovirus infection^26^. To develop human anti-hACE2 mAbs, we used AlivaMab mice, which produce chimaeric antibodies consisting of human Fab domains and a murine Fc domain. To maximize the diversity of the anti-hACE2 mAbs, the KP AlivaMab mouse strain that generates human Kappa light chain (κ) containing antibodies and the AV Alivamab mouse strain that generates both human Kappa (κ) and Lambda (λ) light chains, were immunized with recombinant hACE2 extracellular domains that were either monomeric or rendered dimeric by fusion to the Fc portion of human IgG1. Hybridomas were generated from mice with sera that inhibited SARS-CoV-2 pseudotyped viruses, and hybridoma supernatants were screened for hACE2-binding mAbs by enzyme-linked immunosorbent assay (ELISA) with hACE2-coated plates (Fig. 1a). Eighty-two hybridomas expressing hACE2-binding mAbs were identified, and ten (1C9H1, 2C12H3, 2F6A6, 2G7A1, 4A12A4, 05B04, 05D06, 05E10, 05G01 and 05H02, Fig. 1a) were selected from these on the basis of potent inhibition of pseudotyped virus infection of Huh-7.5 cells. Chimaeric mAbs were purified from the hybridoma culture supernatants and antiviral activity was reconfirmed using the SARS-CoV-2 pseudotype assay. Also the human Fab variable regions (VH and VL) were sequenced (Figs. 1a,b and Supplementary Tables 1 and 2). Four out of five mAbs from the KP AlivaMab mice, namely 05B04, 05D06, 05E10 and 05G01, shared similar, or identical, complementarity-determining regions (CDRs) (Supplementary Tables 1 and 2). In contrast, the mAbs from AV AlivaMab mice were diverse and originated from distinct germline precursors (Supplementary Tables 1 and 2).

### Human anti-hACE2 mAbs inhibit diverse sarbecoviruses

To generate fully human anti-hACE2 mAbs, the variable domains from the six most potent chimaeric human-mouse mAbs were cloned into a human immunoglobulin-γ1 (IgG1) expression vector carrying substitutions at L234/L235 (LALA) and M428/N434 (LS)^23–25^. Human mAbs were thus generated by co-expression of corresponding heavy and light chains. As 05D06 generated low yields, a hybrid mAb, 05B04LC/05D06HC, was made by co-expression of the light chain of 05B04 and the heavy chain of a clonally related mAb, 05D06.

We tested the ability of the six mAbs to inhibit SARS-CoV-2 (Wuhan-hu-1) spike pseudotyped HIV-1 infection in Huh-7.5 target cells. Each of the mAbs was able to inhibit SARS-CoV-2 (Wuhan-hu-1) pseudotyped virus infection with half maximal inhibitory concentration (IC_50_) values from 7.0 ng ml^−1^ to 89 ng ml^−1^ (Fig. 1b and Extended Data Table 1). The two most potent mAbs, 05H02 and 2G7A1, had similar IC_50_ values to potent spike targeting mAbs and were more than tenfold more potent than a previously reported anti-hACE2 antibody, h11B11, that has murine variable regions grafted onto a human antibody^27^.

The six anti-hACE2 mAbs also inhibited infection by SARS-CoV-2 variant pseudotypes, including Beta, Delta and Omicron (BA.1), with comparable potency (IC_50_ values from 8.2 ng ml^−1^ to 197 ng ml^−1^) (Fig. 1b and Extended Data Table 1). Anti-hACE2 mAb-treated Huh-7.5 cells were also challenged with viruses pseudotyped with spike proteins from SARS-CoV or SARS-related coronaviruses from other mammals, specifically pangolin CoV-GD, pangolin CoV-GX, bat CoV Rs4231 and bat CoV Rs7327. All of the sarbecoviruses tested were inhibited by all six the human anti-hACE2 mAbs, with comparable potencies (IC_50_ values from 3.0 ng ml^−1^ to 140 ng ml^−1^) (Fig. 1b–d and Extended Data Table 1).

**Fig. 2:**
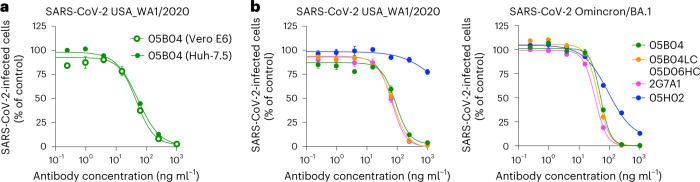
Inhibition of authentic SARS-CoV-2 infection by human anti-hACE2 mAbs. **a**,**b**, Anti-hACE2 mAbs (2G7A1, 05H02, 05B04 and the hybrid antibody 05B04LC/05D06HC) were serially diluted and incubated with Vero E6 and Huh-7.5 target cells (**a**) or Vero E6 target cells only (**b**). Thereafter, cells were infected with authentic SARS-CoV-2 (WA1/2020) (**a** and **b**) or SARS-CoV-2 Omicron BA.1 variants (**b**). Infected cells were quantified by immunostaining and plotted as a percentage of the number of cells infected in the absence of anti-hACE2 mAbs. Mean and range of three independent titrations is plotted. Source data

**Extended Data Fig. 2:**
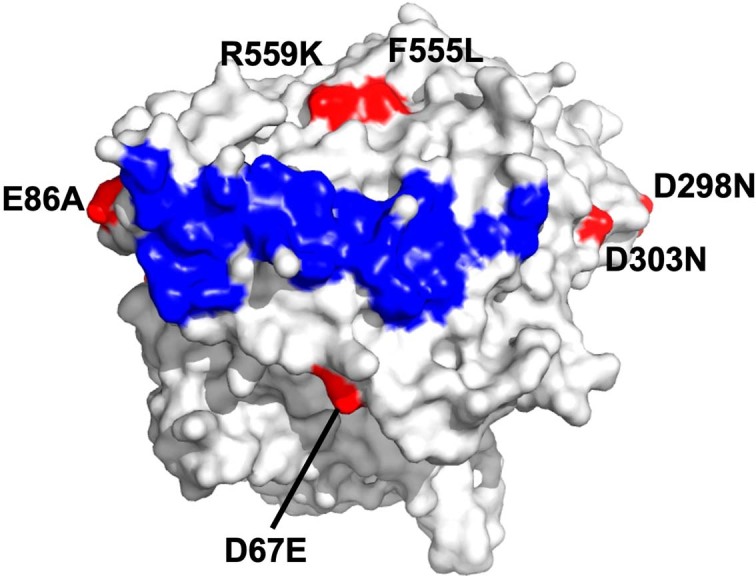
Differences in hACE2 and agmACE2 proximal to the SARS-CoV-2 spike binding site. Structure of the hACE2 is shown (PDB 6LZG), with the SARS-CoV-2 spike binding site highlighted in blue and positions that differ in agmACE2 highlighted in red.

Next, human Huh-7.5 or African green monkey (agm) Vero E6 cells were incubated with the 05B04 mAb and challenged with authentic SARS-CoV-2 USA_WA/2020. 05B04 inhibited infection with similar potency on both cell lines (Fig. 2a). Because SARS-CoV-2 Omicron/BA.1 replicates poorly in Huh-7.5 cells we tested the four most potent antibodies using Vero E6 target cells only. Three mAbs, namely 2G7A1, 05B04 and 05B04LC/05D06HC, potently inhibited both SARS-CoV-2 USA_WA/2020 and SARS-CoV-2 Omicron/BA.1, while 05H02 was less potent in Vero E6 cells (Fig. 2b) than predicted by its activity against spike pseudotypes in Huh-7.5 cells (Extended Data Table 1). Sequence differences between hACE2 and agmACE2 in the vicinity of the RBD binding site might account for the reduced potency of 05H02 in Vero E6 cells (Fig. 2b and Extended Data Fig. 2). Nevertheless, the remaining three human anti-hACE2 mAbs could inhibit infection both by spike pseudotypes and replication-competent SARS-CoV-2.

### Human anti-hACE2 mAbs inhibit SARS-CoV-2 spike–hACE2 binding

**Fig. 3:**
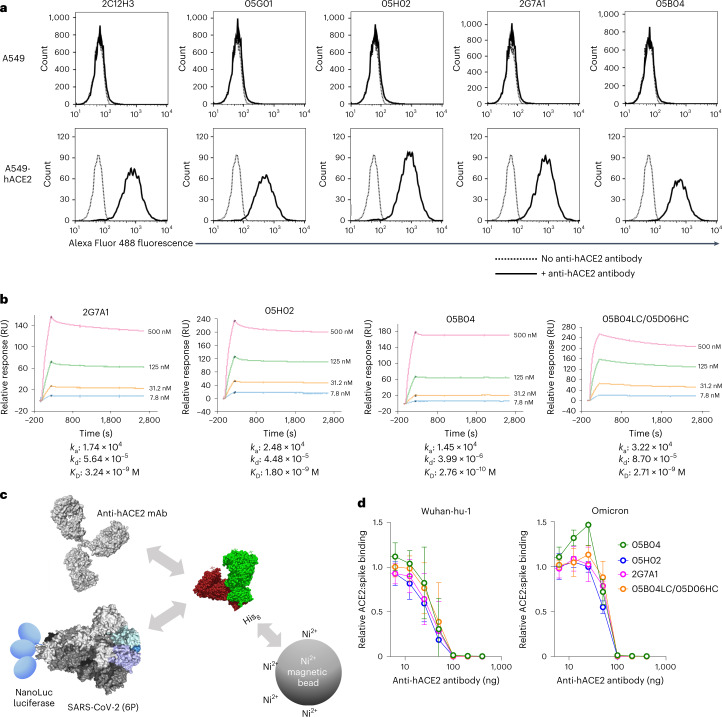
Binding to hACE2 by human anti-hACE2 antibodies. **a**, Parental A549 cells (top) or A549 cells stably expressing hACE2 (bottom) were incubated in the presence (solid lines) or absence (dotted lines) of the indicated hACE2 antibodies. The cells were then incubated with Alexa Fluor 488 conjugated goat anti-human IgG and then analysed by flow cytometry. **b**, Four anti-hACE2 mAbs (2G7A1, 05H02, 05B04 and the hybrid antibody 05B04LC/05D06HC) were immobilized onto a Protein G Sensor chip. His-tagged hACE2 1–740aa proteins (7.8 nM, 31.2 nM, 125 nM or 500 nM) were injected at 30 µl min^−1^ for 240 s followed by a dissociation phase of 2,400 s at a flow rate of 30 µl min^−1^. *K*_D_ values were calculated from the ratio of association and dissociation constants (*K*_D_ = *k*_d_/*k*_a_), derived using a 1:1 binding model. RU, resonance unit. **c**, Schematic representation of the spike–hACE2 binding assay in which NanoLuc luciferase was appended to the C-termini of a conformationally stabilized SARS-CoV-2 spike trimer, S-6P-NanoLuc (based on Wuhan-hu-1, or Omicron BA.1 variants). The fusion protein was incubated with His-tagged hACE2 (1–740aa) that was pre-incubated in the presence or absence of anti-hACE2 mAbs, and complexes were captured using His-tag magnetic beads. **d**, Serially diluted mAbs (2G7A1, 05B04, 05H02 or 05B04LC/05D06HC) were mixed with 100 ng of His-tagged hACE2 1–740aa proteins. After incubation, the mixture was then incubated with Wuhan-hu-1 or Omicron S-6P-NanoLuc proteins (10 ng) followed by capture on His-tag magnetic beads. Bound NanoLuc activity was measured after washing. Mean and range of four independent experiments is plotted. Source data

**Extended Data Fig. 3:**
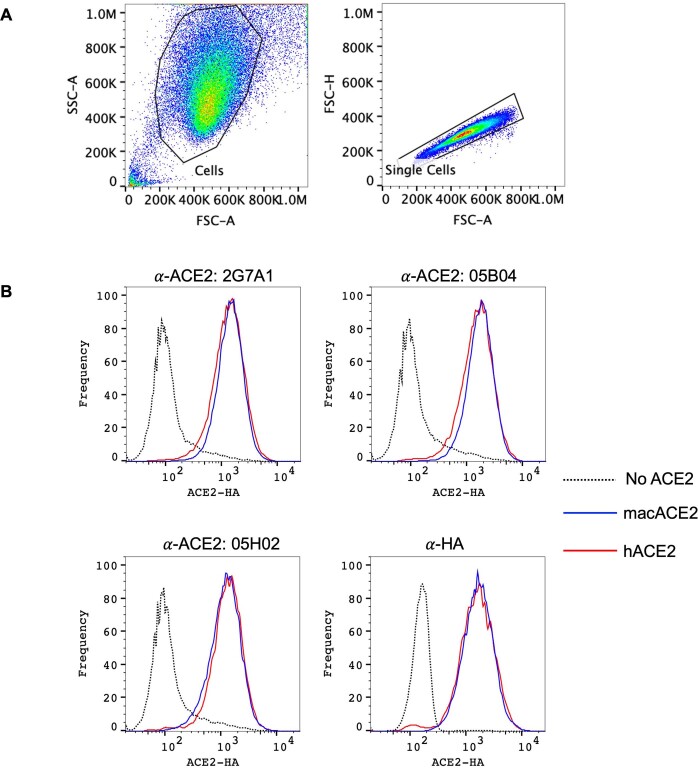
Binding of anti-hACE2 antibodies to human and macaque ACE2. (A) FACS sequential gating strategy for detection of ACE2 on cell surface of A549 cells by human anti-hACE2 antibodies. Applies to Fig. 3A and Extended Data Fig. 3B (B) Parental A549 cells (dashed lines), or A549 cells stably expressing HA-tagged macaque ACE2 (blue, macACE2), or HA-tagged human ACE2 (red, hACE2) were incubated in the presence of the indicated human anti-hACE2 antibodies or anti-HA antibody. The cells were then incubated with Alexa Fluor 488 conjugated goat anti-human IgG (for anti-hACE2 antibodies) or Alexa Fluor 488 goat anti-mouse IgG (for anti-HA antibody) and then analyzed by flow cytometry.

We assessed the interaction between the five non-hybrid human anti-hACE2 mAbs and hACE2 using flow cytometry, where each mAb tested bound to A549 cells expressing hACE2, but not parental A549 cells (Fig. 3a and Extended Data Fig. 3a). Each of the three most potent mAb tested bound to A549 cells expressing macaque ACE2 and hACE2 equivalently (Extended Data Fig. 3a,b). This property should facilitate the pre-clinical evaluation of the mAbs in macaque models.

Affinity measurement using surface plasmon resonance (SPR) revealed association constants between the four most potent mAbs and hACE2 ranging from 1.45 × 10^4^ to 3.22 × 10^4^, while the dissociation constants ranged from 3.99 × 10^−6^ to 4.48 × 10^−5^ (Fig. 3b). As such, the equilibrium dissociation constant (*K*_D_) values ranged from 3.24 × 10^−9^ M to 2.76 × 10^−10^ M (Fig. 3b). These measurements may underestimate the affinity of bivalent mAbs binding to dimeric cell-surface hACE2 due to the use of soluble, monomeric hACE2 to detect binding to immobilized anti-hACE2 mAbs.

To assess inhibition of hACE2:spike binding by anti-hACE2 mAbs, we fused NanoLuc luciferase at the C-terminus of ‘HexaPro’ trimeric SARS-CoV-2 spike (S-6P-NanoLuc) and performed binding assays using immobilized His-tagged hACE2 (Fig. 3c). Pre-incubation of immobilized hACE2 with the anti-hACE2 mAbs blocked hACE2 binding to SARS-CoV-2 (Wuhan-Hu-1) trimers (Fig. 3d). The mAbs also inhibited Omicron BA.1 spike trimer binding to immobilized hACE2 with similar potency (Fig. 3d). Overall, we conclude that human anti-hACE2 mAbs block interactions between hACE2 and SARS-CoV-2 spike and consequently inhibit virus infection with breadth and potency.

### hACE2 genetic variation and mAb binding efficacy

**Extended Data Fig. 4:**
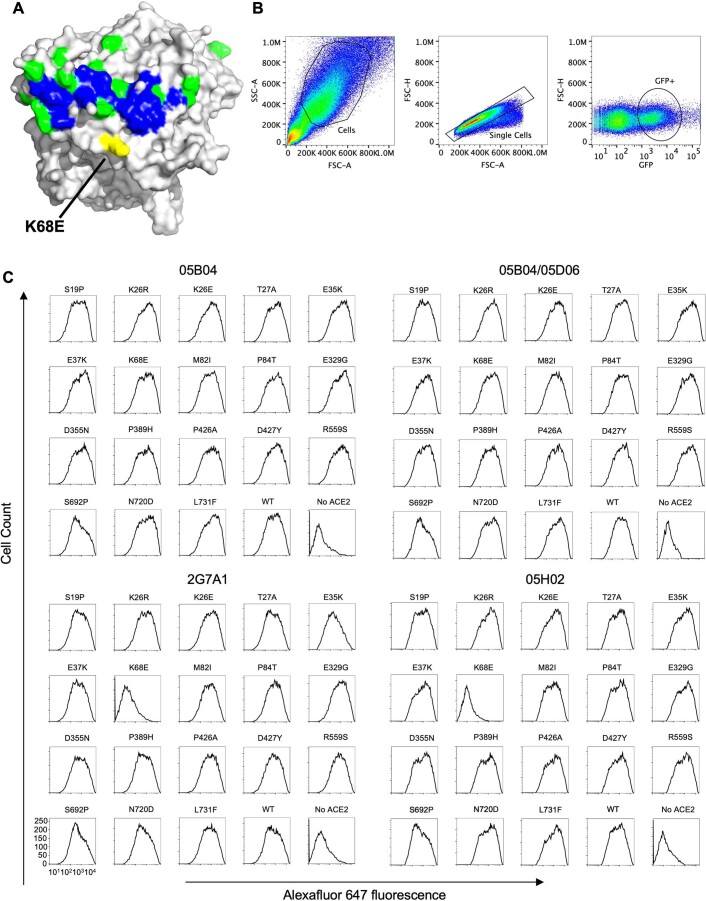
Impact of hACE2 variation on human anti-hACE2 binding. (A) Structure of the hACE2 is shown (PDB 6LZG), with the SARS-CoV-2 spike binding site highlighted in blue and positions that differ in salient hACE2 variants highlighted in green (K68E highlighted in yellow). (B) FACS sequential gating strategy for detection of ACE2 on 293 T cell surface by human anti-hACE2 antibodies. Applies to Extended Data Fig. 4C. (C) FACS analysis of 293 T cells transfected with plasmids expressing eighteen hACE2 variants, wild-type hACE2 (WT) or no hACE2. Cells were stained with anti-hACE2 antibodies as indicated followed by incubation with AlexaFluor 647 conjugated goat anti-human IgG.

Population variation in hACE2 might affect the antiviral efficacy of hACE2-binding mAbs. Non-synonymous hACE2 variants in the vicinity of the SARS-CoV-2 spike binding site occur at frequencies of 0.001–0.388% in humans^28^ (Extended Data Fig. 4a). We carried out fluorescence-activated cell sorting analyses using cells expressing hACE2 with each of 18 non-synonymous variants that encode amino acid substitutions close to the SARS-CoV-2 spike binding site (Extended Data Fig. 4b,c). Seventeen of 18 hACE2 variants did not affect recognition by the four mAbs tested. Only the K68E variant, which occurs at an extremely low frequency in human populations (0.001%) (ref. ^28^), impaired binding by 2G7A1 and 05H02 (Extended Data Fig. 4c). Notably, the K68E substitution is proximal to the D67E substitution in agmACE2 (Extended Data Figs. 2 and 4a), providing a possible explanation for the reduced potency of 05H02 in Vero cells (Fig. 2b). Notably, neither K68E nor any other hACE2 variant affected 05B04 or 05B04LC/05D06HC binding (Extended Data Fig. 4c). Thus, variation in hACE2 among humans should have little effect on anti-hACE2 mAb efficacy.

### Structural analyses of the anti-hACE2 mAb 05B04

**Fig. 4:**
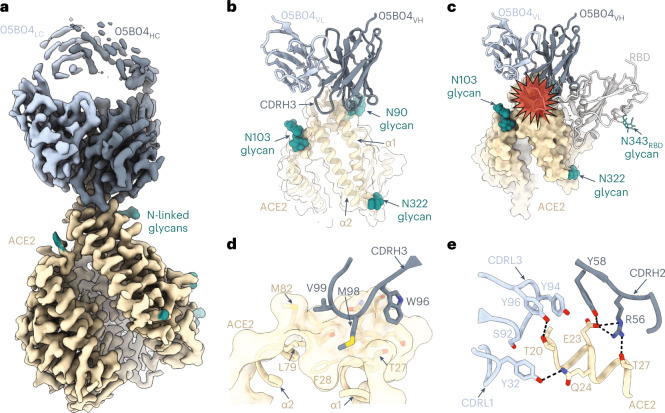
Cryo-EM structure of the 05B04-hACE2 complex. **a**, Cryo-EM density of 3.3 Å for the 05B04 Fab–hACE2 complex. Density for 05B04 Fab variable domains is shown as shades of slate blue, and hACE2 is shown as wheat. **b**, Close-up view of 05B04 variable domains (blue cartoon) binding to hACE2 (surface and cartoon, wheat). N-linked glycans modelled in ACE2 are shown as teal spheres. **c**, Superimposition of PDB 6VW1 on the 05B04-ACE2 structure, aligned on the Cα atoms of the ACE2 α1 and α2 helices. Predicted clashes between the RBD (grey) and 05B04 variable domains (shades of blue) are highlighted with a red star. **d**, CDRH3-mediated contacts on ACE2 (surface and cartoon rendering), which include burying of 05B04 Fab residue Met98_HC_ in a hydrophobic patch between ACE2 α1 and α2 helices. **e**, CDRH2-, CDRL1- and CDRL3-mediated contacts with the α1 helix. Potential hydrogen bonds are shown as black dashed lines.

**Extended Data Fig. 5:**
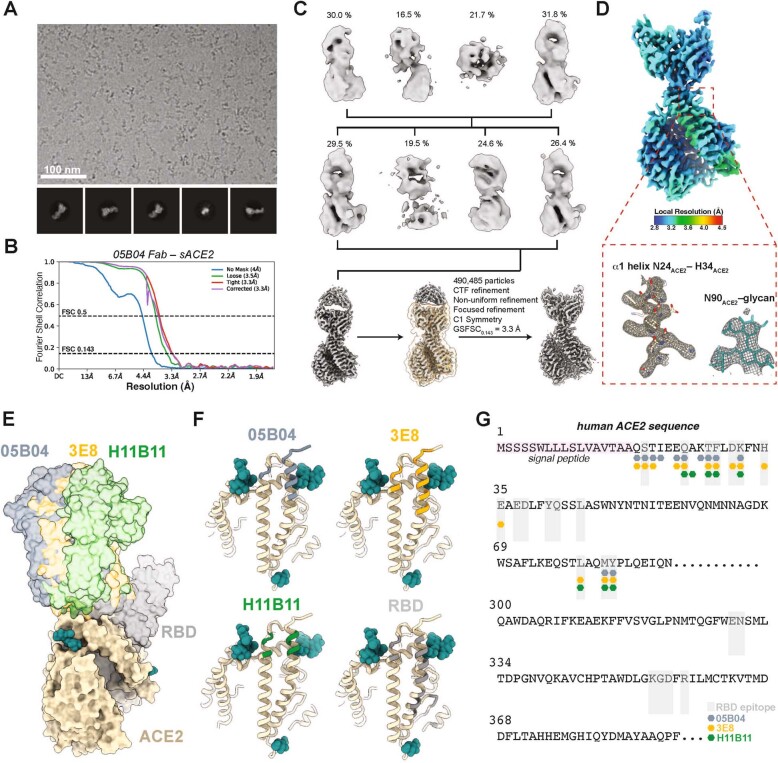
Cryo-EM data processing, validation, and structure comparison. (A) Representative micrograph of 4,907 used and 2D class averages for 05B04-hACE2 data collection. (B) Gold-standard FSC plot calculated in cryoSPARC for local refinement. (C) 3D classification and refinement logic tree for the 05B04-hACE2 cryo-EM data set. (D) Local resolution estimate for 05B04-hACE2 locally refined map and representative cryo-EM density contoured at 7σ for boxed regions. (E) Structural superposition of anti-hACE2 mAbs 05B04 (slate blue), 3E8 (orange), h11B11 (green) and SARS-CoV-2 RBD (gray) modeled on soluble ACE2 (wheat). (F, G) Comparison of anti-hACE2 mAb epitopes visualized on an ACE2 model (F) or highlighted on the ACE2 sequence (G). anti-hACE2 mAb epitopes are colored slate blue, orange, and green for 05B04, 3E8, and h11B11, respectively. The SARS-CoV-2 RBD epitope is denoted as shaded gray boxes (G).

To delineate the structural basis for broad neutralization of anti-hACE2 mAbs, we determined the structure of soluble hACE2 (ref. ^29^) bound to the antigen-binding fragment (Fab) of 05B04, which is among the most potent mAbs whose binding was not impaired by naturally occurring human ACE2 variation, using single-particle cryo-EM. Focused refinement resulted in a 3.3 Å resolution map, revealing a 05B04 Fab bound to the N-terminal helices of hACE2 (Fig. 4a,b, Extended Data Fig. 5a–d and Supplementary Table 3). The binding orientation of 05B04 sterically hinders and competes with SARS-CoV-2 RBD (Fig. 4c) and is similar to the binding orientations and epitopes of murine antibodies 3E8 and h11B11 (Extended Data Fig. 5e–g)^26,27^. The 05B04 CDR loops CDRH2, CDRH3, CDRL1 and CDRL3 contribute to binding an epitope that comprises residues from the α1 and α2 helices, resulting in a total buried surface area (BSA) of ~1,265 Å^2^ (613 Å^2^ epitope BSA + 652 Å^2^ paratope BSA). The CDRH3 loop mediates extensive polar and van der Waals contacts, including insertion of Met98_HC_ into a hydrophobic pocket involving Phe28, Leu79, Met82 and Tyr83 of hACE2 (Fig. 4d). CDRH2, CDRL1 and CDRL3 loops contribute to a hydrogen bond network with both main chain and side chain atoms of hACE2. For example, Gln24 of hACE2 forms a hydrogen bond with the hydroxyl group of Tyr32_LC_, while hACE2 residue Glu23 forms a salt bridge with Arg56_HC_ (Fig. 4e). These interactions mimic favourable interactions between the SARS-CoV-2 RBD ridge and the N-terminal α1 helix of hACE2, which were critical to increases in affinity for SARS-CoV-2 RBD relative to SARS-CoV RBD^30,31^. Thus, 05B04-mediated inhibition of ACE2-binding sarbecoviruses is achieved through molecular mimicry of SARS-CoV-2 RBD interactions, providing high binding affinity to hACE2 despite the smaller binding footprint on hACE2 relative to the RBD (Extended Data Fig. 5f,g). These structural analyses are likely to be generally similar for the 05B04LC/05D06HC hybrid antibody but different for 05H02 and 2G7A1 whose binding is sensitive to the K68E substitution, which is located outside the 050B4 binding site.

### Anti-hACE2 mAbs and human hACE2 enzymatic activity

**Fig. 5:**
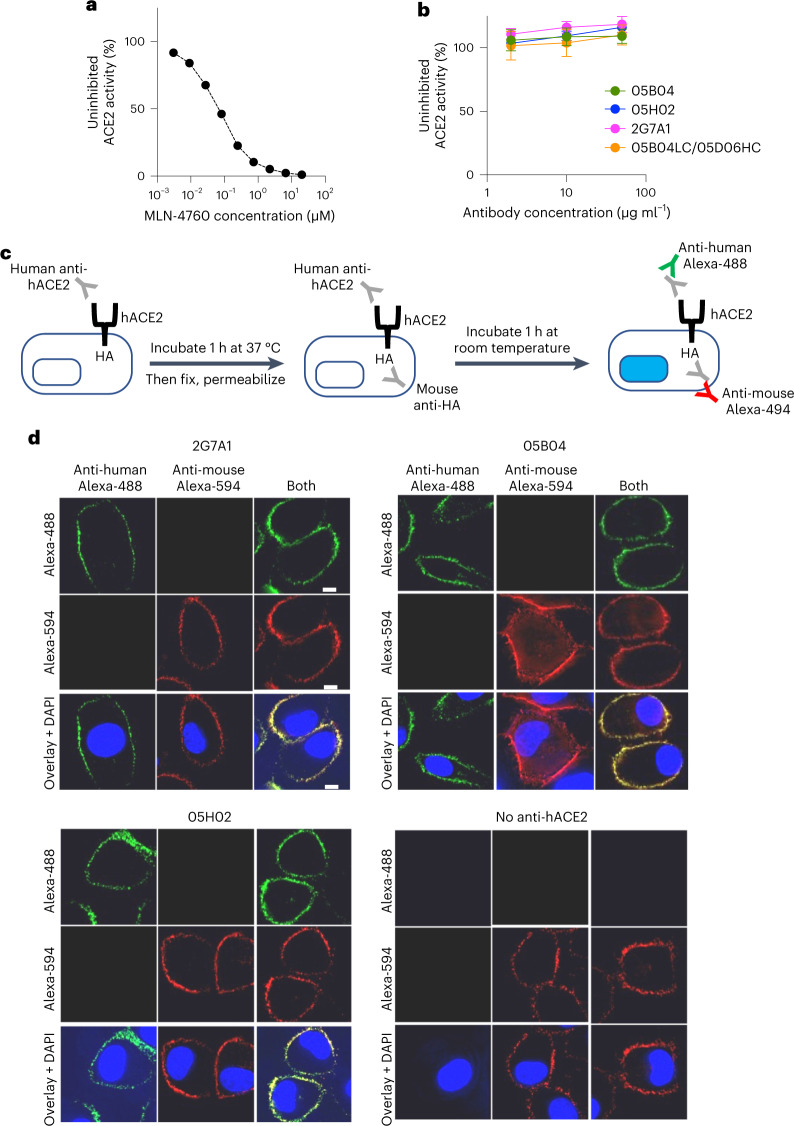
No effect of anti-hACE2 mAbs on hACE2 activity and distribution. **a**,**b**, The ACE2 inhibitor control MLN-4670 (**a**) or anti-hACE2 mAbs (2G7A1, 05B04, 05H02 and 05B04LC/05D06HC, 2 μg ml^−1^, 10 μg ml^−1^ and 50 μg ml^−1^) (**b**) were mixed with hACE2 (0.2 μg ml^−1^) and a fluorogenic ACE2 substrate in 96-well plates. After incubation, fluorescence intensity (555 nm/585 nm, excitation/emission), indicative of hACE2 enzymatic activity, was measured and plotted as a percentage of the uninhibited control. Mean and range of four independent experiments is plotted. **c**, Outline of an assay to determine hACE2 and anti-hACE2 mAb internalization. Live A549 cells expressing a C-terminally (intracellular) HA-tagged hACE2 receptor were incubated with anti-hACE2 mAbs, Then, cells were fixed and permeabilized. Total hACE2-HA was then immunostained with a mouse anti-HA-tag antibody. The internalization of hACE2-HA and anti-hACE2 mAbs was then evaluated by staining with anti-mouse Alexa Fluor 594 (Alexa-594) and goat anti-human Alexa Fluor 488 (Alexa-488), respectively. **d**, Localization of anti-hACE2 mAbs (green, left) or hACE2-HA (red, centre) or both (right) following incubation of live A549/hACE2-HA cells with 2G7A1, 05B04 or 05H02, or the absence of human anti-hACE2 antibody as indicated. Blue stain (DAPI) indicates cell nuclei. Scale bars, 10 μm. Representative of three independent experiments. Source data

ACE2 catalyses the hydrolysis of angiotensin I or angiotensin II (refs. ^32,33^). Its active site is distal to the N-terminal α1 helix, where sarbecovirus spike proteins and 05B04 bind^34,35^ (Extended Data Fig. 5e–g). To measure any effect on hACE2 activity, the anti-hACE2 mAbs were mixed with hACE2 protein at varying molar ratios (10:1, 50:1 or 250:1) and hACE2 enzymatic activity was determined. While the inhibitor MLN-4760 inhibited hACE2 activity at concentrations of 10–100 nM (Fig. 5a), none of the anti-hACE2 mAbs tested affected hACE2 activity (Fig. 5b). Thus, while allosteric inhibition of hACE2 activity by the mAbs was theoretically possible, such inhibition did not occur.

**Extended Data Fig. 6:**
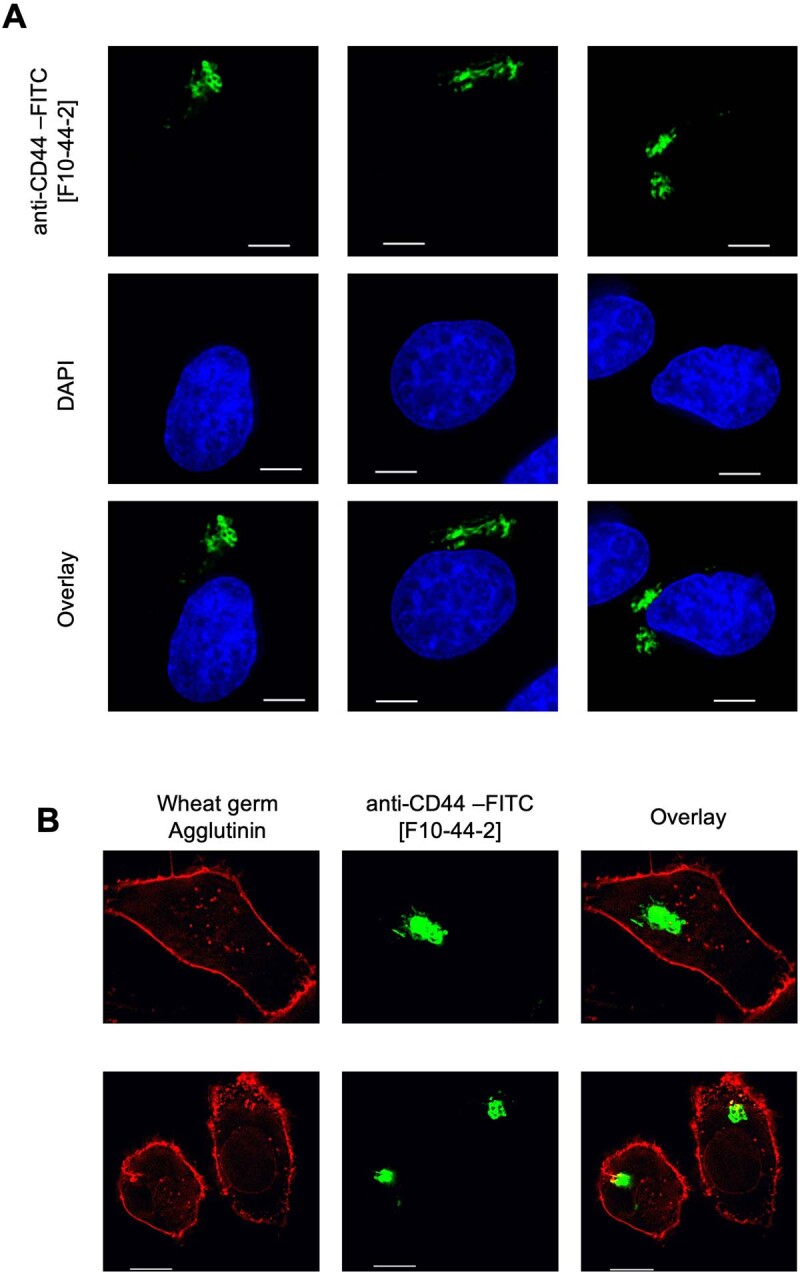
Internalization of a control anti-CD44 antibody in A549/hACE2-HA cells. (A, B) Localization of anti-CD44 antibody (green, upper panels) following incubation of live A549/hACE2-HA cells with FITC-labelled anti-CD44 [F10-44-2]. Blue stain (DAPI) indicates cell nuclei in panel (A) where three representative fields are shown. Red indicates staining of cell surface with Wheat Germ agglutinin conjugated with Alexa Fluor™ 594 in panel B where two representative fields are shown. Scale bar: 10 μm. Representative of two experiments. Source data

To test whether anti-hACE2 mAbs binding induced hACE2 downregulation from the cell surface, we developed a fluorescence-based assay in which anti-hACE2 mAbs were incubated with A549 cells expressing hACE2, influenza haemagglutinin (HA, amino acids 98–106)-tagged at its intracellular C-terminus. Following mAb incubation, cells were fixed and stained to reveal the subcellular distribution of the anti-hACE2 mAbs and the HA-tagged hACE2 (Fig. 5c). As a control, cells were incubated with an anti-CD44 antibody, which is internalized in A549 cells^36^. Each anti-hACE2 mAb tested exhibited near complete co-localization with hACE2-HA marked by anti-HA antibody staining. Both hACE2 protein and the anti-hACE2 mAbs remained localized on the cell surface, while the control anti-CD44 antibody accumulated at intracellular sites (Fig. 5d and Extended Data Fig. 6a,b). Only very low levels of intracellular hACE2 were detected, irrespective of mAb treatment. Thus, the anti-hACE2 mAbs had no effect on hACE2 internalization or recycling, suggesting that the anti-hACE2 mAbs would be unlikely to undergo accelerated target-dependent clearance from the circulation during in vivo use.

### Anti-hACE2 mAbs protect against SARS-CoV-2 infection in mice

We next determined whether our set of four human anti-hACE2 mAbs could protect against SARS-CoV-2 infection in an animal model. We used hACE2 knock-in mice that are susceptible to SARS-CoV-2 infection^37^, in which the endogenous mouse ACE2 is replaced by hACE2 and thereby more likely mimic the levels and distribution of hACE2 expression encountered in humans.

**Fig. 6:**
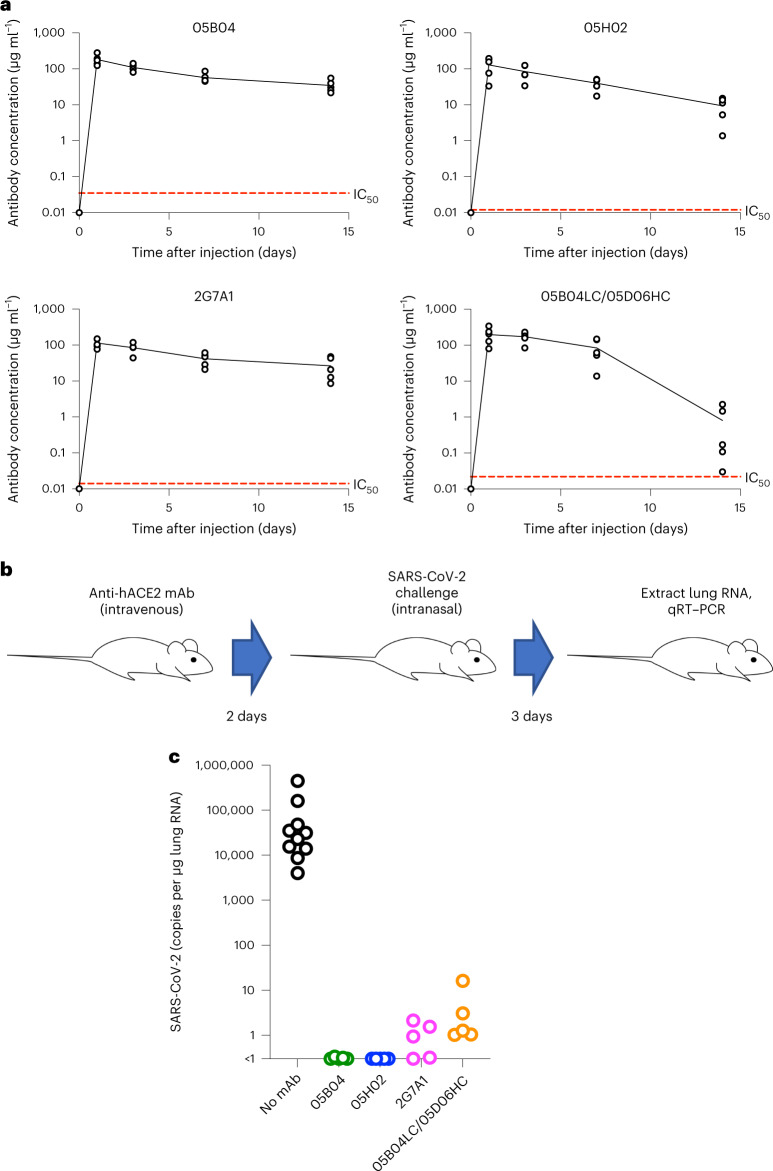
Prophylaxis of human anti-hACE2 antibodies for SARS-CoV-2 infection in mice. **a**, Anti-hACE2 mAb levels following subcutaneous injection of 250 μg (equivalent to approximately 12.5 mg kg^−1^) of anti-hACE2 antibodies (05B04, 05H02, 2G7A1 and 05B04LC/05D06HC) into each of five hACE2 knock-in mice (B6.129S2(Cg)-Ace2tm1(ACE2)Dwnt/J) on day 0. Serum dilutions (0.5 μl, 0.1 μl or 0.02 μl) of the sera collected on the indicated days or mAb standards were mixed with hACE2 (1–740aa)-NanoLuc fusion protein and captured by Protein G beads (Extended Data Fig. 4). Dashed red line indicates IC_50_ for inhibition of SARS-CoV-2 pseudotyped virus infection. **b**,**c**, Human ACE2 knock-in mice (B6.129S2(Cg)-Ace2tm1(ACE2)Dwnt/J) (*n* = 5) were injected with 250 μg (equivalent to 12.5 mg kg^−1^) of the anti-hACE2 antibodies (05B04, 05H02, 2G7A1 or 05B04LC/05D06HC): at 2 days after antibody injection mice were challenged intranasally with SARS-CoV-2, USA_WA/2020 P3, 2 × 10^5^ PFU per mouse (virus titres measured on Vero E6 cells) (**b**); 3 days later, mouse lungs were collected and RNA extracted, and the number of viral genomes per µg of total lung RNA was measured using qRT–PCR (**c**). Source data

**Extended Data Fig. 7:**
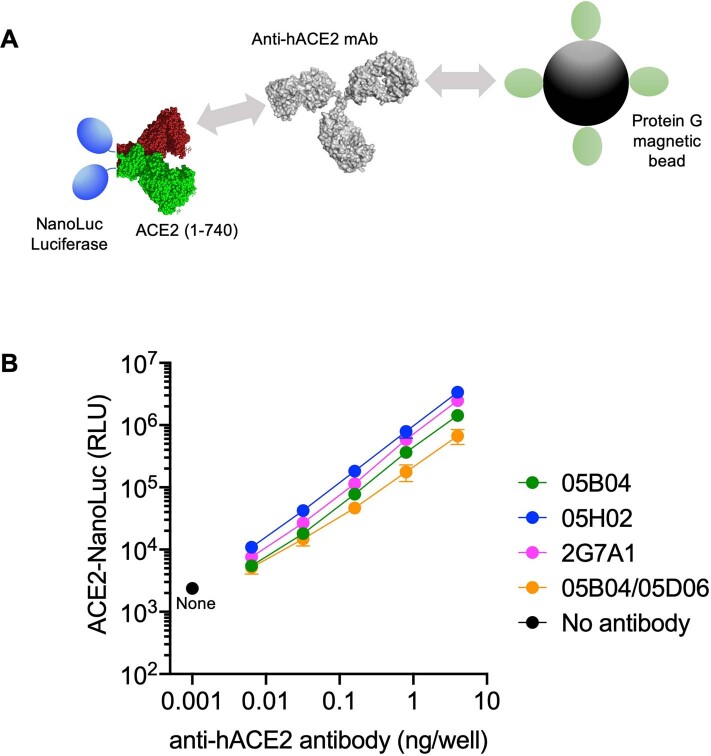
Determination of anti-hACE2 antibody levels using hACE2-NanoLuc fusion protein. (A) Schematic representation of the NanoLuc assay in which NanoLuc luciferase was appended to the C-termini of the hACE2 extracellular domain (1-740 aa). The fusion protein was incubated with anti-hACE2 mAbs and complexes were then captured using Dynabeads™ Protein G magnetic beads. (B) For the standard curves, 40 ng of the indicated anti-hACE2 mAbs and five-fold serial dilutions thereof, were mixed with 30 ng of hACE2(1-740 aa)-NanoLuc protein. After 1 hr incubation at 4 °C, the mixture was incubated with Dynabeads™ Protein G magnetic beads. The beads were washed and bound NanoLuc activity was measured. Mean and range of four independent experiments is plotted. Source data

First, we determined the pharmacokinetic behaviour of the hACE2 mAbs. To measure the levels of anti-hACE2 mAb in mouse serum, we generated a hACE2 (1–740)-NanoLuc fusion protein that could be captured by protein G magnetic beads when bound to anti-hACE2 mAbs (Extended Data Fig. 7a). This assay allowed the detection of ~0.01 ng to 10 ng of anti-hACE2 mAb in mouse serum, with linear standard curves in this concentration range (Extended Data Fig. 7b). Mice were injected subcutaneously with 250 μg of each anti-hACE2 mAb (~12.5 mg kg^−1^) on day 0, and the levels of mAbs in serum were measured until day 14. For 05B04, 05H02 and 2G7A1, the serum mAb concentration remained above 10 μg ml^−1^ on day 14 (Fig. 6a), 100–1,000-fold higher than their IC_50_ values. For 05B04LC/05D06 HC, the mAb levels remained stable for around 7 days but dropped from day 7 to 14, suggestive of the onset of an immune response to the non-self human antibody. The mean serum half-life for 05B04, 05H02 and 2G7A1 over 14 days was 8.3 days, 5.3 days and 9.6 days, similar to that typically observed for human IgG1 in mice^38^. Overall, the anti-hACE2 mAbs showed favourable pharmacokinetics and conferred no obvious ill effects on the hACE2 knock-in mice.

Each of four anti-hACE2 mAbs was then injected intravenously into mice (five mice per group) 2 days before intranasal challenge with 2 × 10^5^ plaque forming units (PFU) of SARS-CoV-2, strain USA_WA/2020 P3 (Fig. 6b). Levels of viral RNA in the lungs of mock treated mice were in the range of 10^4^–10^6^ copies per μg total RNA at 2 days after infection, comparable to previous observations using this model^37^. Strikingly, pre-treatment of the mice with any one of the four anti-hACE2 mAbs, reduced SARS-CoV-2 replication in lungs, to levels below or close to the limit of detection (~1 copy of viral RNA per μg total RNA) (Fig. 6c). Thus, when used prophylactically in hACE2 knock-in mice, the anti-hACE2 mAbs provided near-sterilizing protection against lung SARS-CoV-2 infection.

### Resilience of anti-hACE2 mAbs to SARS-CoV-2 escape

**Extended Data Fig. 8:**
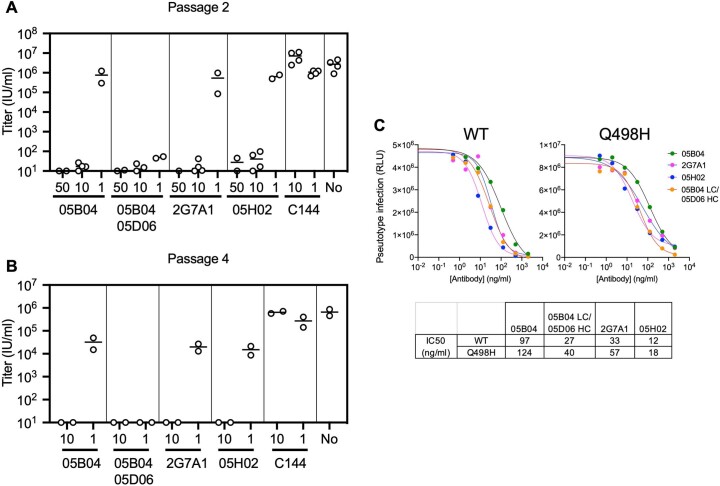
Sensitivity to anti-hACE2 antibodies after rVSV/SARS-CoV-2/GFP replication in the presence of anti-hACE2 antibodies. (A, B) Infectious virus yield after rVSV/SARS-CoV-2/GFP replication for 2 passages (A) or 4 passages (B) in the presence of each of four anti-hACE2 antibodies, or the anti-RBD antibody C144. The concentrations (in μg/ml) indicated on the X-axis or in the absence of antibody (No). Data are derived from two independent experiments. (C) Inhibition of HIV-1 based pseudotyped virus infection by anti-hACE2 mAbs. Anti-hACE2 antibodies (05H02, 2G7A1, 05B04 and the hybrid antibody 05B04 LC/05D06 HC) were incubated with Huh-7.5 target cells and cells were infected with pseudotypes bearing WT or Q498H spike proteins. Infection was measured using NanoLuc luciferase assays. Mean of two independent titrations is plotted, and IC_50_ values for each anti-hACE2 antibody virus combination is given below. Source data

To assess whether SARS-CoV-2 spike can evolve to evade our set of anti-hACE2 mAbs, we used a recombinant virus carrying SARS-CoV-2 spike (rVSV/SARS-CoV-2/GFP)^39^ that rapidly produces SARS-CoV-2 escape mutants that are resistant to anti-spike mAbs^10,11^. Diversified populations of 10^6^ infectious units of rVSV/SARS-CoV-2/GFP were applied to 293T/ACE2cl.22 cells that express high levels of ACE2 (ref. ^39^), pre-treated with varying anti-hACE2 mAb concentrations (1 to 50 μg ml^−1^), and passaged up to four times in the presence of each mAb (Extended Data Fig. 8a,b). A control anti-spike antibody (C144, IC_50_ <10 ng ml^−1^) selected rVSV/SARS-CoV-2/GFP variants that replicated at titres equivalent to the no antibody controls within two passages (Extended Data Fig. 8a,b) and carried RBD substitutions (E484K or Q493R) that abolish C144 sensitivity, in line with previous findings^10^. Conversely, 10 or 50 μg ml^−1^ of the anti-hACE2 mAbs blocked replication and rVSV/SARS-CoV-2/GFP was undetectable after passage 4 (Extended Data Fig. 8a,b). At reduced concentration (1 μg ml^−1^), replication was extinguished by 05B04LC/05D06HC and reduced but not extinguished for 05B04, 2G7A1 and 05H02 (Extended Data Fig. 8a,b). Spike sequences amplified from rVSV/SARS-CoV-2/GFP at passage 4 with 1 μg ml^−1^ 05B04 or 2G7A1 revealed no changes, while one of two replicate rVSV/SARS-CoV-2/GFP cultures with 05H02 harboured a Q498H substitution. Interestingly, Q498 substitutions arose during SARS-CoV-2 adaptation to rodents^40,41^ and increase spike affinity for hACE2 (ref. ^42^), which could plausibly reduce the susceptibility to anti-hACE2 mAbs. Nevertheless, Q498H conferred only minor (<2-fold) increased IC_50_ for the anti-hACE2 mAbs in pseudotype infection assays (Extended Data Fig. 8c). We conclude that the barrier to the acquisition of resistance to anti-hACE2 antibodies by SARS-CoV-2 is high.

## Discussion

As SARS-CoV-2 has adapted to the immunological pressures imposed by naturally elicited spike-binding antibodies, many therapeutic mAbs have become obsolete^13^. Although it might be possible to isolate additional spike-targeting human mAbs, SARS-CoV-2 will continue to evolve and adapt to human antibodies which will probably limit broad utility of any new spike-targeting mAb therapy. As we report here, human anti-hACE2 mAbs offer the chance of durable, ‘resistance proof’ prophylaxis and treatment. Although SARS-CoV-2 might acquire the ability to use alternate receptors^43^, such an event would represent a much greater genetic hurdle to overcome than evasion of spike-targeting mAbs.

ACE2 has a role in the regulation of cardiovasular and renal function^32,44^. However, the hACE2 enzymatic active site is distal to the site that is bound by sarbecovirus spike proteins^34,35^ and the anti-hACE2 mAbs we report here. Indeed, none of the four most potent mAbs affected hACE2 enzymatic activity and none induced the internalization of hACE2 that is normally localized on the cell surface. Thus, the mAbs are not predicted to have deleterious side effects based on their target specificity.

While the current work suggests that anti ACE2 antibodies will be effective inhibitors of sarbecovirus infection in humans, the fact that the antibodies target a host receptor molecule rather than the viral spike protein will necessitate the testing of safety, efficacy and pharmacological behaviour of anti-ACE2 antibodies in primate models before human clinical trials. Ultimately, anti-hACE2 mAbs may be particularly useful in patients who are especially vulnerable, for example those with immunodeficiency or who are undergoing immunosuppressive treatment and in which vaccine-elicited protective immunity is more difficult to achieve^45–47^. mAbs with ‘LS’ mutations that enhance interaction with the neonatal Fc receptor and prolong antibody half-life^23–25^ may enable a single protective dose during the early phase of high incidence waves or seasons. Moreover, the exceptional breadth and potency with which our engineered mAbs inhibit infection by hACE2-utilizing sarbecoviruses might mean that they could be an effective intervention in the event of future outbreaks of SARS-like coronaviruses.

## Methods

### Cell lines

Human embryonic kidney HEK-293T cells (American Type Culture Collection (ATCC) CRL-3216) and the derivative expressing hACE2, that is, 293T/hACE2.cl22 (ref. ^39^), Caco-2 cells (ATCC HTB-37), human hepatoma-derived Huh-7.5 cells^48^, Vero E6 cells and a derivative expressing TMPRSS2 (ref. ^49^) and A549 cells (adenocarcinomic human alveolar basal epithelial cells) were maintained in Dulbecco’s modified Eagle medium (DMEM) supplemented with 10% foetal bovine serum (Sigma F8067) and gentamycin (Gibco). All cell lines used in this study were monitored periodically to ensure the absence of retroviral contamination and mycoplasma.

### Generation of hACE2-specific human mAbs

ACE2-binding mAbs with human variable regions were generated using AlivaMab Mouse (Ablexis) transgenic mouse strains. Specifically, AlivaMab mice were immunized subcutaneously with recombinant human ACE2 extracellular domain (1–740aa) fused to human IgG1 Fc and/or a polyhistidine tag. Mice were immunized at 3 week intervals at least four times, using 10 µg subcutaneous injections at different sites. Mice with sera exhibiting SARS-CoV-2 infection inhibiting activity in the pseudotype virus assay were acute boosted before fusion of splenocytes with SP2/0 cells for hybridoma generation. Hybridomas expressing antibodies that bound to hACE2 were identified by ELISA using plates coated with purified hACE2. Anti-hACE2 hybridoma supernatants that contained antibodies with human ACE2 binding activity were tested for inhibition of SARS-CoV-2 pseudotyped virus infection of Huh-7.5 cells. This analysis indicated ten hybridoma antibodies that were positive for binding and potently inhibited SARS-CoV-2 pseudotype virus infection that were chosen for hybridoma cell subcloning and expansion. Antibodies were purified from these hybridoma culture supernatants and were further tested for potency ranking in the SARS-CoV-2 pseudotype virus inhibition assay.

### Human mAb expression plasmids

DNA encoding the variable regions of the heavy (VH) and light (VL) from hybridomas was PCR amplified from DNA extracted from the hybridoma cell lines. For the 05B04 and 05D06 antibodies, the DNA sequences encoding VH and VL were human codon-optimized using GenSmart Codon Optimization, synthesized by Integrated DNA Technologies (IDT). For each mAb, DNA encoding VH were fused to complementary DNA encoding the Fc domain of human IgG1, in which Fc domain was modified to include the substitutions at L234 L235 (LALA) that abolish FcR–gamma interaction, and substitutions at M428 N434 (LS) that enhance interaction with the neonatal Fc receptor to prolong mAb half-life in humans^23–25^. To construct the expression plasmids for heavy and light chain antibody expression^50^, PCR amplicons or synthetic DNA encoding variable regions were subcloned using AgeI and XhoI (for LC), or AgeI and SalI (for HC), respectively, using NEBuilder HiFi DNA Assembly.

### Human ACE2 and sarbecovirus spike expression plasmids

Plasmids expressing the spike proteins from SARS-CoV, SARS-CoV-2 (Wuhan-hu-1, Beta (B.1.351), Delta (B.1.617.2) and Omicron (B.1.1.529) variants), the pangolin (*Manis javanica*) coronaviruses from Guangdong, China (pCoV-GD) and Guanxi, China (pCoV-GX) were previously described^14,39,51^. Human codon-optimized cDNAs encoding spike proteins from the rufous horseshoe bat (*Rhinolophus sinicus*) coronaviruses Rs4231 and Rs7327 were generated using GenSmart Codon Optimization, synthesized by IDT as gBlocks, and inserted into the pCR3.1 expression vector using NheI and XbaI and NEBuilder HiFi DNA Assembly.

To construct the plasmids expressing NanoLuc-fused to conformationally stabilized versions of the SARS-CoV-2 Wuhan-hu-1 or Omicron variant spike proteins, the HexaPro (6P) modified cDNA was fused at its C-terminus with DNA encoding a trimerization domain, a GGSGG spacer sequence, NanoLuc luciferase (NLuc), a human rhinovirus 3C protease cleavage site and a polyhistidine tag (8XHis). This cDNA, termed (S-6P-NanoLuc), was inserted into the pCR3.1 expression vector.

To construct a plasmid expressing catalytically inactive His-tagged or IgG1 Fc-fused soluble ectodomain of hACE2 (1–740aa), H374N and H378N substitutions were introduced by overlap extension PCR into a hACE2 cDNA and a His-tag was fused to its C-terminus, and the purified PCR product was inserted into the pCAGGS expression vector (hACE2-1–740aa-His). To construct the expression plasmid encoding hACE2 (1–740aa)-NanoLuc-8XHis, the hACE2 1-740aa and NanoLuc-8XHis fragments were PCR amplified using hACE2-1–740aa-His and S-6P-NanoLuc as templates, respectively, followed by Gibson assembly and insertion into the pCR3.1 expression vector. Oligonucleotide sequences used during molecular construction are provided in Supplementary Data 1.

### Protein and antibody expression and purification

To express the monomeric, His-tagged human ACE2 extracellular domain (residues 1–740) used as immunogen, His-tagged hACE2 (1–740aa)-NanoLuc or soluble hACE2 used for cryo-EM studies (residues 1–614), Expi293 cells were transfected with the expression plasmid hACE2-1–740aa-8xHis, hACE2(1–740aa)-NanoLuc-8XHis, or hACE2-1–614aa-8xHis using ExpiFectamine 293 (Thermo Fisher Scientific), respectively. Four days later, the supernatant was filtered with 0.22 μm membrane filter and loaded on Ni-NTA agarose (Qiagen) and, after washing, hACE2 proteins were eluted with 200 mM imidazole in phosphate-buffered saline (PBS). For cryo-EM structural studies, a subsequent size-exclusion chromatography step on a Superdex 200 10/300 column (Cytiva) was performed against PBS, and fractions corresponding to monomeric soluble hACE2 were pooled and stored at 4 °C. Dimeric, Fc-fused hACE2 extracellular domain was also expressed in Expi293 cells in the same way. The secreted proteins in supernatant were first incubated with Protein G Sepharose 4 Fast Flow overnight at 4 °C, loaded into column and, after washing, eluted with 0.1 M glycine, pH 2.9 into tubes containing 1/10th volume of 1 M Tris, pH 8.0.

To express mAbs, Expi293 cells were transfected with the corresponding light chain and heavy chain expression plasmids at the ratio of 1:1 using ExpiFectamine 293. Four days later, the mAbs in the supernatant were purified through Protein G Sepharose 4 Fast Flow and eluted with 0.1 M glycine, pH 2.9 as described above.

To express S-6P-NanoLuc proteins, Expi293 cells were transfected with S-6P-NanoLuc expression plasmids that included for the original Wuhan-hu-1 or Omicron spike variants using ExpiFectamine 293. Three days later, the supernatant was collected and loaded on Ni-NTA agarose and, after thorough wash, S-6P-NanoLuc proteins were released after HRV 3C protease (TaKaRa) treatment overnight at 4 °C.

All recombinant proteins, including purified mAbs, were dialysed against PBS before used in further experiments.

### Sarbecovirus spike-bearing pseudotypes and infectivity inhibition assay

To generate HIV-1 virions pseudotyped with sarbecovirus spikes, including SARS-CoV, SARS-CoV-2 (Wuhan-hu-1, Beta, Delta and Omicron variants), pangolin coronavirus pCoV-GD, pangolin coronavirus pCoV-GX and bat coronaviruses SARSr-CoV 4231 and SARSr-CoV 7327, ten million 293T cells in a 15 cm dish were transfected with 25 μg of an HIV-1 envelope-deficient proviral plasmid expressing NanoLuc along with 7.5 μg of spike expression plasmids, in which the C terminal 19aa was truncated (Δ19) (ref. ^39^). Cells were washed twice with PBS the next morning, and virions were collected at 48 h post transfection, filtered (0.22 μm) and purified by Lenti-X Concentrator (TaKaRa). To measure the infectivity, viral stocks were twofold serially diluted and added to Huh-7.5 cells^39^, which express hACE2 (ref. ^52^) and are permissive to SARS-CoV-2 (ref. ^53^), in 96-well plates seeded 1 day before infection. Cells were then collected at 48 h post infection for measuring NanoLuc activity using the Nano-Glo Luciferase Assay System and GloMax Navigator Microplate Luminometer (Promega).

To measure antiviral activity, the hACE2 mAbs were fourfold serially diluted (beginning with 2 μg ml^−1^) in 96-well plates over seven dilutions and incubated with Huh-7.5 target cells for 1 h at 37 °C. Thereafter, the mAb-treated Huh-7.5 cells were infected with sarbecovirus spike pseudotyped viruses. Cells were collected 48 h post infection and NanoLuc luciferase activity measured in infected cells as described above.

### SARS-CoV-2 virus stocks and titration

SARS-CoV-2 strains USA-WA1/2020 and the Omicron variant B.1.1.529 were obtained from BEI Resources (catalogue nos. NR-52281 and NR-56461, respectively). The original virus (WA1/2020) was amplified in Caco-2 cells, which were infected at a multiplicity of infection of 0.05 PFU per cell and incubated for 5 days at 37 °C. The Omicron variant B.1.1.529 was amplified in Vero E6 cells (ATCC) that were engineered to stably express TMPRSS2. Vero-TMPRSS2 cells were infected at a multiplicity of infection of 0.05 PFU per cell and incubated for 4 days at 33 °C. Virus-containing supernatants were subsequently collected, clarified by centrifugation (3,000*g* × 10 min), filtered with a 0.22 μm membrane and stored at −80 °C. To measure the virus stock titres by standard plaque assay, 500 µl of serial tenfold virus dilutions in Opti-MEM were used to infect 4 × 10^5^ Vero E6 cells (from Ralph Baric) in six-well plates. After 1.5 h adsorption, the virus inoculum was removed, and cells were overlaid with DMEM containing 10% FBS with 1.2% microcrystalline cellulose (Avicel). Cells were incubated for 4 days at 33 °C, followed by fixation with 7% formaldehyde and crystal violet staining for plaque enumeration. All SARS-CoV-2 experiments were performed in a biosafety level 3 laboratory.

### SARS-CoV-2 inhibition assays

The day before infection, Vero E6/Huh-7.5 cells were seeded at 1 × 10^4^ cells per well into 96-well plates. Antibodies were serially diluted in DMEM, mixed with target cells and incubated for 60 min at 37 °C. Subsequently a constant amount of SARS-CoV-2 was added to achieve 40–50% virus-positive cells. Cells were fixed 18–24 h after infection by adding an equal volume of 7% formaldehyde to the wells, followed by permeabilization with 0.1% Triton X-100 for 10 min. After extensive washing, cells were incubated for 1–2 h at room temperature with blocking solution of 5% goat serum in PBS (005-000-121; Jackson ImmunoResearch). A rabbit polyclonal anti-SARS-CoV-2 nucleocapsid antibody (GTX135357; GeneTex) was added to the cells at 1:1,000 dilution in blocking solution and incubated at 4 °C overnight. A goat anti-rabbit Alexa Fluor 594 (A-11012; Life Technologies) at a dilution of 1:2,500 was used as a secondary antibody. Nuclei were stained with Hoechst 33342 (62249; Thermo Scientific) at a concentration of 1 μg ml^−1^. Images were acquired with a fluorescence microscope and analysed using ImageXpress Micro XLS and MetaXpress software (Molecular Devices).

### MAb binding measurements using SPR

SPR experiments were performed using a Biacore 8K instrument (GE Healthcare). Human mAbs 2G7A1, 05B04, 05H02 and hybrid mAb 05B04LC/05D06HC were captured with a Series S Sensor ship Protein G (Cytiva) at a concentration of 20 nM at a flow rate of 10 μl min^−1^ for 60 s. Flow cell 1 was kept empty and used as a negative control. A concentration series of His-tagged hACE2 1–740aa proteins (fourfold dilutions from a maximum concentration of 500 nM) was injected at 30 µl min^−1^ for 240 s followed by a dissociation phase of 2,400 s at a flow rate of 30 µl min^−1^. Binding reactions were allowed to reach equilibrium, and *K*_D_ values were calculated from the ratio of association and dissociation constants (*K*_D_ = *k*_d_/*k*_a_), which were derived using a 1:1 binding model that was globally fit to all curves in a dataset. Flow cells were regenerated with 10 mM glycine pH 1.5 at a flow rate of 30 μl min^−1^ for 30 s.

### Spike hACE2 binding and binding inhibition assay

To evaluate whether anti-hACE2 mAbs inhibit spike–hACE2 interaction, 400 ng of each mAb (2G7A1, 05B04, 05H02 or 05B04LC/05D06HC) and a twofold serial dilution thereof over seven dilutions were mixed with 100 ng of His-tagged hACE2 1-740aa proteins in PBS containing 2% bovine serum albumin. After 1 h incubation at 4 °C, the mixture was incubated with 10 ng of Wuhan-1 or Omicron S-6P-NanoLuc proteins for 1 h at 4 °C. Then 1 μl of Dynabeads His-Tag Isolation and Pulldown magnetic beads (Thermo Fisher Scientific) was added into each well. After 1 h incubation at 4 °C, the beads were washed three times and bound NanoLuc activity measured using Nano-Glo Luciferase Assay System and a GloMax Navigator Microplate Luminometer (Promega).

### Cryo-EM sample preparation, data collection and structure refinement

Purified 05B04 Fab was mixed with monomeric soluble hACE2 (residues 1–614) at an equimolar concentration for 1 h at room temperature. Fab-hACE2 complex was concentrated to 4 mg ml^−1^ and deposited on a freshly glow discharged 300 mesh, R1.2/1.3 Quantifoil grid (Electron Microscopy Sciences). Samples were vitrified in 100% liquid ethane using a Mark IV Vitrobot (Thermo Fisher) after blotting at room temperature and 100% humidity for 3 s with Grade 595 filter paper (Ted Pella).

Single-particle cryo-EM data were collected on a Titan Krios transmission electron microscope equipped with a Gatan K3 direct detector, operating at 300 kV and controlled using SerialEm automated data collection software^54^. A total dose of 60 e^−^ Å^−2^ was accumulated on each movie comprising 40 frames with a pixel size of 0.867 Å and a defocus range of −1.0 to −2.6 µm. Data processing was carried out as previously described^49^ using cryoSPARC v3.2 (ref. ^55^) and summarized in Supplementary Table 3. Briefly, 6,599 movies were patch motion corrected for beam-induced motion and contrast transfer function (CTF) estimates were performed on non-dose weighted micrographs in cryoSPARC v3.2. After curation to remove images with poor CTF fits and ice contamination, particles were automatically picked using blob picker, extracted 4x-binned and 2D classified. Class averages that showed secondary structure features were pooled and used to generate ab initio volumes (*n* = 4). Particles corresponding to volumes that resembled the Fab–sACE2 complex were re-extracted at 2x-bin, pooled and subsequently 3D classified in cryoSPARC. Particles corresponding to the 3D classes with well-defined features were pooled, unbinned and subjected to CTF and non-uniform refinement (C1 symmetry) in cryoSPARC. This resulted in a global map with an estimated resolution of 3.5 Å based on gold standard FSC calculations. A mask was generated to exclude Fab constant domains from focused refinement, which yielded an overall map resolution for the locally refined volume of 3.3 Å as calculated using the gold-standard Fourier shell correlation of 0.143 criterion.

### Structure modelling, refinement and analysis

Coordinates for initial complexes were generated by docking individual chains from reference structures into cryo-EM density using UCSF Chimera^56^. An initial model for the 05B04 Fab–hACE2 structure was generated from coordinates of Protein Data Bank (PDB) 7S0B (chain A: 05B04 heavy chain), PDB 7DPM (chain B: 05B04 light chain) and PDB 6VW1 (chain A: hACE2). Models were refined using one round of rigid body refinement with morphing followed by real space refinement in Phenix^57^. Sequence-updated models were built manually in Coot^58^ and then refined using iterative rounds of refinement in Coot and Phenix. Glycans were modelled at potential N-linked glycosylation sites in Coot. Validation of model coordinates was performed using MolProbity^59^.

Structure figures were made with UCSF ChimeraX^60^. Local resolution maps were calculated using cryoSPARC v3.2. BSAs were calculated using PDBePISA^61^ and a 1.4 Å probe. Potential hydrogen bonds were assigned as interactions that were <4.0 Å and with A–D–H angle >90°. Potential van der Waals interactions between atoms were assigned as interactions that were <4.0 Å. Hydrogen bond and van der Waals interaction assignments are tentative due to resolution limitations. hACE2 epitope residues were defined as residues containing atom(s) within 4.5 Å of a 05B04 Fab atom.

### hACE2 enzymatic activity assay

To measure the effect of anti-hACE2 mAbs on the catalytic activity of hACE2, various concentrations of 2G7A1, 05B04, 05H02 and 05B04LC/05D06HC (2 μg ml^−1^, 10 μg ml^−1^ and 50 μg ml^−1^) were mixed with hACE2 at 0.2 μg ml^−1^. Human ACE2 enzymatic activity was then measured using the ACE2 Inhibitor Screening Assay Kit (BPS Bioscience) following the manufacturer’s instructions. The intensity of the fluorescent product of the hACE2 reaction product was detected at 555 nm/585 nm (excitation/emission) with Clariostar Plus Microplate Reader (BMG Labtech). MLN-4760 (Sigma, #5306160001) served as a positive control ACE2 inhibitor.

### Flow cytometric analysis of cell surface hACE2 binding by human anti-hACE2 mAbs

To evaluate the ability of anti-hACE2 mAbs to bind to cell surface ACE2 by flow cytometry, A549 cells (human alveolar basal epithelial cells) were engineered to express ACE2. The hACE2 or macaque (mac)ACE2-expressing cells were detached from plates with 10 mM ethylenediaminetetraacetic acid in PBS and 10^5^ cells incubated in the absence or the presence of human anti-hACE2 mAbs (2 μg ml^−1^) for 2 h at 4 °C. After washing, the cells were incubated with Alexa Fluor 488 conjugated goat anti-human IgG (Thermo Fisher Scientific). Flow cytometry was performed using Attune NxT Acoustic Focusing Cytometer (Thermo Fisher Scientific, V3.1.2 and V5.1.0). The same procedure was applied to parental, unmodified A549 cells as a negative control for non-specific cell surface binding.

To assess the impact of hACE2 variation on the interaction between hACE2 and human anti-hACE2 antibodies, human 293T cells were transfected with plasmids expressing 18 hACE2 variants, including S19P, K26R, K26E, T27A, E35K, E37K, K68E, M82I, P84T, E329G, D355N, P389H, P426A, D427Y, R559S, S692P, N720D, L731F and wild-type hACE2, respectively, along a GFP-expressing plasmid. Cells transfected with plasmids carrying GFP only served as the negative control. Two days later, cells were collected and stained with anti-hACE2 antibodies followed by incubation with Alexa Fluor 647 conjugated goat anti-human IgG (Thermo Fisher Scientific). GFP-positive populations were gated to measure anti-hACE2 mAbs binding.

### hACE2 internalization assay

To determine whether the anti-hACE2 mAbs induced hACE2 internalization, live A549 cells expressing hACE2 receptor with an HA-epitope tag appended to its intracellular C-terminus were incubated with anti-hACE2 mAbs (1 μg ml^−1^) for 1 h at 37 °C. Then, cells were fixed with 4% paraformaldehyde/PBS, treated with 10 mM glycine and permeabilized with 0.1% Triton X-100. Total hACE2-HA was then detected with mouse anti-HA.11 antibody (BioLegend, cat 901503, clone 16B12, 1 μg ml^−1^). The internalization of the hACE2-HA protein and anti-hACE2 mAbs was then evaluated by staining with goat anti-mouse Alexa Fluor 594 (to detect the HA-tagged hACE2) (1/500 dilution) and/or goat anti-human Alexa Fluor 488 antibodies (to detect the anti-hACE2 mAbs) (1/500 dilution) (Thermo Fisher Scientific). As a control, anti-CD44 antibody conjugated with FITC (clone F10-44-2 from abcam, cat. no. ab30405, 1/20 dilution) was used. Wheat germ agglutinin conjugated with Alexa Fluor 594 (Thermo Fisher Scientific, cat. no. W11262, 5 μg ml^−1^) were used to visualize the cell surface. Images were captured using a DeltaVision OMX SR imaging system (GE Healthcare).

### Analysis of anti-hACE2 mAbs pharmacokinetics in mice

Six-week-old hACE2-knock-in female mice, in which human ACE2 cDNA replaces the endogenous mouse ACE2 sequences, were obtained from Jackson Labs (B6.129S2(Cg)-Ace2tm1(ACE2)Dwnt/J, strain 035000). The mice were housed at a temperature of 22 °C and a humidity of 30–70% under a 12 h–12 h light–dark cycle with ad libitum access to food and water. After acclimatization for 2 weeks, these mice received subcutaneous injections of 250 μg of human anti-hACE2 mAbs per mouse (*n* = 5). Mice were bled on day 0, day 1, day 3, day 7 and day 14 with blood collected into Microvette CB 300 Serum (Sarstedt).

Serially diluted mouse plasma (fivefold serial dilution over four dilutions from a maximum volume of 0.5 μl) was diluted in PBS buffer containing 2% bovine serum albumin and mixed with 30 ng of His-tagged hACE2(1-740aa)-NanoLuc protein. After 1 h incubation at 4 °C, the mixture was incubated with 3 μl of Dynabeads Protein G magnetic beads (Thermo Fisher Scientific). After 1 h rotation at 4 °C, the beads were washed three times and bound NanoLuc activity was measured using Nano-Glo Luciferase Assay System and a GloMax Navigator Microplate Luminometer (Promega). To construct standard calibration curves for measurement of mAb levels in plasma, 100 ng of mAbs (2G7A1, 05B04, 05H02 or 05B04LC/05D06HC) were fivefold serially diluted over seven dilutions and mixed with hACE2(1-740aa)-NanoLuc proteins. MAb:hACE2(1-740aa)-NanoLuc complexes were captured and quantified in parallel with those formed using the plasma samples from mAb-infused mice.

All of the animal procedures and experiments were performed by following protocols approved by the Rockefeller University Institutional Animal Care and Use Committee.

### SARS-CoV-2 challenge experiments in hACE2-expressing mice

Six-week-old hACE2-knock-in female mice, in which human ACE2 cDNA replaces the endogenous mouse ACE2 sequences, were obtained from Jackson Labs (B6.129S2(Cg)-Ace2tm1(ACE2)Dwnt/J, strain 035000). The mice were housed at a temperature of 22 °C and a humidity of 30–70% under a 12 h–12 h light–dark cycle with ad libitum access to food and water. After acclimatization for 2 weeks, the mice (five mice per treatment group) were injected retro-orbitally with 250 μg (equivalent to ~12.5 mg kg^−1^) of anti-hACE2 mAbs. At 2 days after mAb injection, mice were challenged intranasally with SARS-CoV-2, USA_WA/2020 P3, 2 × 10^5^ PFU per mouse (virus titres determined on Vero E6 cells). At 3 days after infection, mouse lungs were dissected and homogenized in TRIzol. Chloroform was added to induce phase separation. Then after centrifugation, RNA in the aqueous phase was precipitated with isopropanol and, after wash with ice-cold 75% ethanol, dissolved in nuclease-free water. The number of viral genomes per microgram of total lung RNA was measured by qRT–PCR, using Power SYBR Green RNA-to-CT 1-Step Kit (Thermo Fisher Scientific) on StepOne Plus Real-Time PCR system (Applied Biosystems). The primers used target RNA sequences encoding the nucleocapsid protein: 2019-nCoV_N1-F: 5′-GACCCCAAAATCAGCGAAAT-3′ and 2019-nCoV_N1-R: 5′-TCTGGTTACTGCCAGTTGAATCTG-3′. The standard was obtained from IDT (2019-nCoV_N_Positive Control 10006625).

Infection experiments in mice, and all procedures involved therein, were approved by the Rockefeller University Institutional Animal Care and Use Committee.

### Selection of rVSV/SARS-CoV-2 variants in the presence of antibodies

HEK-293T/ACE2cl.22 cells were incubated with anti-hACE2 mAbs at concentrations of 50, 10 or 1 μg ml^−1^ for 1 h at 37 °C. Viral populations (rVSV/SARS-CoV-2/GFP_2E1_) containing 1 × 10^6^ infectious units were then added to the cells. As a control, the rVSV/SARS-CoV-2/GFP_2E1_ populations were incubated with the spike-specific mAb C144 (10 or 1 μg ml^−1^) for 1 h before infection. After 24 h, the medium was replaced with fresh medium containing the respective concentration of each mAb. After another 24 h, the virus-containing supernatant was filtered (0.22 μm), and 100 μl of the supernatant was added to HEK-293T/ACE2cl.22 cells, which had been pre-incubated with anti-ACE2 mAb (50, 10 or 1 μg ml^−1^) for 1 h at 37 °C as indicated above. Two passages at 50 μg ml^−1^ were or four passages at 10 and 1 μg ml^−1^ were carried out. RNA was extracted from 100 μl of filtered p4 supernatant and reverse transcribed using the SuperScript VILO cDNA Synthesis Kit (Thermo Fisher Scientific). Sequences encoding the extracellular domain of the spike protein were amplified using KOD Xtreme Hot Start Polymerase (Sigma-Aldrich, 719753). These PCR products were sequenced to identify escape substitutions.

### Reporting summary

Further information on research design is available in the Nature Portfolio Reporting Summary linked to this article.

### Supplementary information

Supplementary Information Supplementary Table 1. CDR sequences of anti-hACE2 mAb heavy chains. Supplementary Table 2. CDR sequences of anti-hACE2 mAb light chains. Supplementary Table 3. Cryo-EM data collection, refinement and validation statistics for 05B04 Fab–hACE2 1–614 complex.

Reporting Summary

Supplementary Data 1 Sequences of oligonucleotides used in molecular construction.

### Source data

Source Data Fig. 1 Statistical source data.

Source Data Fig. 2 Statistical source data.

Source Data Fig. 3 Statistical source data.

Source Data Fig. 5 Statistical source data and unprocessed images.

Source Data Fig. 6 Statistical source data.

Source Data Extended Data Fig. 1 Statistical source data.

Source Data Extended Data Fig. 6 Unprocessed images.

Source Data Extended Data Fig. 7 Statistical source data.

Source Data Extended Data Fig. 8 Statistical source data.

## Figures and Tables

**Extended Data Table 1:**
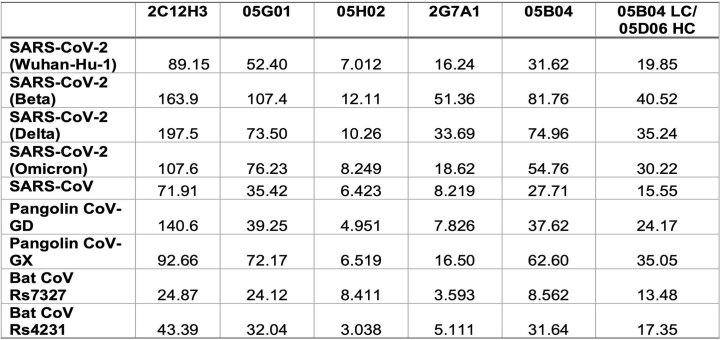
Antiviral potency (IC_50_ values, ng/ml) of fully human anti-hACE2 mAbs

IC_50_ values for anti-hACE2 mAb inhibition of pseudotyped virus infection determined using HIV-1 based pseudoviruses and Huh-7.5 target cells, as described in Fig. 1.

## Data Availability

The atomic model and cryo-EM map generated for the 05B04-hACE2 complex have been deposited at the Protein Data Bank (PDB) (http://www.rcsb.org/) and the Electron Microscopy Databank (EMDB) (http://www.emdataresource.org/) under accession codes 8E7M and EMD-27939, respectively. All other numerical data are in the accompanying source data files and have been deposited with Figshare (10.6084/m9.figshare.22572892).
