# Supplementary material for: Pan-sarbecovirus prophylaxis with human anti-ACE2 monoclonal antibodies
Source: Nat Microbiol. 2023 May 15;8(6):1051–63. doi: 10.1038/s41564-023-01389-9 (PMC10234812; doi:10.1038/s41564-023-01389-9)
Supplement: Supplementary file 1 — Supplementary Table 1. CDR sequences of anti-hACE2 mAb heavy chains. Supplementary Table 2. CDR sequences of anti-hACE2 mAb light chains. Supplementary Table 3. Cryo-EM data collection, refinement and validation statistics for 05B04 Fab–hACE2 1–614 complex. [file 41564_2023_1389_MOESM1_ESM.pdf]

nature microbiology

Article

https://doi.org/10.1038/s41564-023-01389-9

Pan-sarbecovirus prophylaxis with human anti-ACE2 monoclonal antibodies

In the format provided by the authors and unedited

Supplementary information

Table S1: CDR sequences of anti-hACE2 mAb heavy chains

	Alleles	CDR1	CDR2	CDR3
05B04	IGHV3-23*01	GFTFSSYA	ISGSGDRT	AKDWAMVGADAFDI
05D06	IGHV3-23*01	GFTFSSYA	ISISGGST	VKDWYIVGADAFDI
05E10	IGHV3-23*01	GFTFSSYA	INISGGST	VKDWYIMGADAFDI
05G01	IGHV3-23*01	GFTFSNYA	ISINGDRT	AKDWAIVGADAFDV
05H02	IGHV3-23*01	GFTFNIYA	FSGSRYNT	AKEAVAGQFDY
2G7A1	IGHV4-59*01	DGSISGYY	IHYSGTT	ARGPVFWYFDL
2F6A6	IGHV4-59*01	DGSISGYY	IYYSGTT	ARGPVFWYFDL
4A12A4	IGHV3-21*07	GFTFSSYS	ITNGINNI	ARYYYYYGMDV
2C12H3	IGHV4-4*07	GGSITSYY	IFSSGIT	ARHDGFGWFDP
1C9H1	IGHV2-5*01	GFSLSTPGVA	IFWNDDE	AHLALYWYFDF

Table S2: CDR sequences of anti-hACE2 mAb light chains

	Alleles	CDR1	CDR2	CDR3
05B04	IGKV1-17*03	QGISNY	AAS	LQHSYYPYT
05D06	IGKV1-17*03	QGISNY	ATS	LQHSNYPYT
05E10	IGKV1-17*03	QGISNY	ATS	LQHSNYPYT
05G01	IGKV1-17*03	QVISNY	AGS	LQHNNYPYT
05H02	IGKV1-5*03	QSISSW	KAS	QQYNTYSRT
2G7A1	IGLV6-57*01	SGSINNNY	DDN	QSYDSSSRV
2F6A6	IGLV6-57*01	SGSISNSY	DDN	QSYDSSSRV
4A12A4	IGLV3-19*01	SLRNYY	GKN	NSRDSGGNHVV
2C12H3	IGKV3-20*01	QSVSSSY	GAS	QQYGSSPRT
1C9H1	IGLV2-23*01	SSDVGSYNL	EGS	CSYAGSNIVV

Table S3. Cryo-EM data collection, refinement, and validation statistics for 05B04 Fab - hACE2 1-614 complex

**PDB**	**8E7M**
**EMD**	**27939**
**Data collection conditions**	
Microscope	Titan Krios
Camera	Gatan K3 Summit
Magnification	130,000x
Voltage (kV)	300
Dose rate (e^-/pixel/s)	17.6
Electron dose (e^-/Å^2)	60
Defocus range (µm)	1.0-2.6
Pixel size (Å)	0.8677
Micrographs collected	6,599
Micrographs used	4,907
Total extracted particles	2,055,266
Final refined particles	490,485
Symmetry imposed	C1
Nominal Map Resolution (Å), FSC=0.143	
Global Map (unmasked/masked)	3.9/3.5
Focused Map (unmasked/masked)	3.7/3.3
**Refinement and Validation**	
Initial model	6VW1, 7S0B, 7DPM
Model Resolution (Å)	
FSC 0.143	3.3
Number of atoms	
Protein	8,028
Ligand	146
MapCC (global/local)	0.79/0.75
Map sharpening B-factor	112.6
R.m.s. deviations	
Bond lengths (Å)	0.004
Bond angles (˚)	0.814
MolProbity score	1.74
Clashscore (all atom)	7.89
Poor rotamers (%)	0
Ramachandran plot	
Favored (%)	95.5
Allowed (%)	4.5
Disallowed (%)	0
