# Supplementary material for: Pan-sarbecovirus prophylaxis with human anti-ACE2 monoclonal antibodies
Source: Nat Microbiol. 2023 May 15;8(6):1051–63. doi: 10.1038/s41564-023-01389-9 (PMC10234812; doi:10.1038/s41564-023-01389-9)
Supplement: Supplementary file 2 — Reporting Summary [file 41564_2023_1389_MOESM2_ESM.pdf]

nature portfolio

Corresponding author(s): Paul Bieniasz

Last updated by author(s): April 4, 2023

Reporting Summary

Nature Portfolio wishes to improve the reproducibility of the work that we publish. This form provides structure for consistency and transparency in reporting. For further information on Nature Portfolio policies, see our Editorial Policies and the Editorial Policy Checklist.

Statistics

For all statistical analyses, confirm that the following items are present in the figure legend, table legend, main text, or Methods section.

n/a	Confirmed	
☐	☒	The exact sample size (*n*) for each experimental group/condition, given as a discrete number and unit of measurement
☐	☒	A statement on whether measurements were taken from distinct samples or whether the same sample was measured repeatedly
☒	☐	The statistical test(s) used AND whether they are one- or two-sided<br>*Only common tests should be described solely by name; describe more complex techniques in the Methods section.*
☒	☐	A description of all covariates tested
☒	☐	A description of any assumptions or corrections, such as tests of normality and adjustment for multiple comparisons
☒	☐	A full description of the statistical parameters including central tendency (e.g. means) or other basic estimates (e.g. regression coefficient) AND variation (e.g. standard deviation) or associated estimates of uncertainty (e.g. confidence intervals)
☒	☐	For null hypothesis testing, the test statistic (e.g. *F*, *t*, *r*) with confidence intervals, effect sizes, degrees of freedom and *P* value noted<br>*Give P values as exact values whenever suitable.*
☒	☐	For Bayesian analysis, information on the choice of priors and Markov chain Monte Carlo settings
☒	☐	For hierarchical and complex designs, identification of the appropriate level for tests and full reporting of outcomes
☒	☐	Estimates of effect sizes (e.g. Cohen's *d*, Pearson's *r*), indicating how they were calculated

Our web collection on statistics for biologists contains articles on many of the points above.

Software and code

Policy information about availability of computer code

Data collection	AttuneTM NxT software V3.1.2 &V5.1.0; OMX Acquire SR; Biacore 8K/2565817, CLARIOstar Plus (BMG LabTech), cryoSPARC v3.2, PDBePISA
Data analysis	FlowJo 10.8.1; softWoRx 7.0.0; Fiji ImageJ 1.52b; Biacore Insight Evaluation 3.0.12.15655

For manuscripts utilizing custom algorithms or software that are central to the research but not yet described in published literature, software must be made available to editors and reviewers. We strongly encourage code deposition in a community repository (e.g. GitHub). See the Nature Portfolio guidelines for submitting code & software for further information.

Data

Policy information about availability of data

All manuscripts must include a data availability statement. This statement should provide the following information, where applicable:

- Accession codes, unique identifiers, or web links for publicly available datasets
- A description of any restrictions on data availability
- For clinical datasets or third party data, please ensure that the statement adheres to our policy

The atomic model and cryo-EM map generated for the 05B04-hACE2 complex has been deposited at the Protein Databank (PDB) (http://www.rcsb.org/) and the Electron Microscopy Databank (EMDB) (http://www.emdataresource.org/) under accession codes 8E7M and EMD-27939, respectively. All other numerical data is in the accompanying source data files and has been deposited with Figshare.

Human research participants

Policy information about studies involving human research participants and Sex and Gender in Research.

Reporting on sex and gender	not applicable
Population characteristics	not applicable
Recruitment	not applicable
Ethics oversight	not applicable

Note that full information on the approval of the study protocol must also be provided in the manuscript.

Field-specific reporting

Please select the one below that is the best fit for your research. If you are not sure, read the appropriate sections before making your selection.

☒ Life sciences ☐ Behavioural & social sciences ☐ Ecological, evolutionary & environmental sciences

For a reference copy of the document with all sections, see nature.com/documents/nr-reporting-summary-flat.pdf

Life sciences study design

All studies must disclose on these points even when the disclosure is negative.

Sample size	There was no basis on which to select sample size (number of replicate experiments) prior to completing the study. Sample size was chosen arbitrarily
Data exclusions	No data were excluded from data analysis.
Replication	All attempts reached the same conclusion, numbers of repetitions varies as as stated in the manuscript.
Randomization	No allocation was involved in this study.
Blinding	The prophylaxis experiments in which the antibodies provide protection against SARS-CoV-2 infection in human ACE2 knock-in mice was blinded experiment, in that one group of scientists administered antibodies while another group measured the viral RNA. Blinded experiments were also done for antibody evaluation, in which one scientist (JJ) made the antibodies and another scientist (FZ) tested their potency. Under some circumstances, a third scientist (TH) performed neutralization assay using live viruses (Fig 2). Other expriments were not blinded as it was impractical and unnecessary to do so

Reporting for specific materials, systems and methods

We require information from authors about some types of materials, experimental systems and methods used in many studies. Here, indicate whether each material, system or method listed is relevant to your study. If you are not sure if a list item applies to your research, read the appropriate section before selecting a response.

Materials & experimental systems

n/a	Involved in the study	
☐	☒	Antibodies
☐	☒	Eukaryotic cell lines
☒	☐	Palaeontology and archaeology
☐	☒	Animals and other organisms
☒	☐	Clinical data
☒	☐	Dual use research of concern

Methods

n/a	Involved in the study	
☒	☐	ChIP-seq
☐	☒	Flow cytometry
☒	☐	MRI-based neuroimaging

Antibodies

Antibodies used	Monoclonal antibodies: anti-HA (BioLegend anti-HA.11, cat 901503, clone 16B12), FITC Anti-CD44 antibody [F10-44-2] (abcam, cat ab30405), and anti-hACE2 monoclonal antibodies including 2G7A1, 05B04, 05D06, 05H02, 1C9H1, 2C12H3, 2F6A6, 4A12A4, 05E10, and 05G01 (developed in this study).

	Polyclonal antibody: SARS-CoV-2 (COVID-19) Nucleocapsid antibody (GeneTex, cat GTX135357).<br><br>Secondary antibodies: goat anti-mouse Alexa Fluor 594 antibody (ThermoFisher Scientific, Cat # A-11005), goat anti-human Alexa Fluor488 antibody (ThermoFisher Scientific, Cat # A-11013), and goat anti-human Alexa Fluo 647 antibody (ThermoFisher Scientific, Cat # A-21445).<br><br>Lectin used in immunofluorescence: Wheat Germ Agglutinin, Alexa Fluor™ 594 Conjugate (ThermoFisher Scientific, cat W11262) (https://www.thermofisher.com/order/catalog/product/W11262).
Validation	anti-HA from BioLegend: tested in western blot (WB), immunocytochemistry (ICC), immunoprecipitation (IP), and flow cytometry (FC) (https://www.biolegend.com/en-us/products/purified-anti-ha-11-epitope-tag-antibody-11374).<br>FITC Anti-CD44 antibody [F10-44-2] from abcam: tested in flow cytometry (FC) (https://www.abcam.com/fitc-cd44-antibody-f10-44-2-ab30405.html).<br>SARS-CoV-2 (COVID-19) Nucleocapsid antibody (GeneTex, cat GTX135357): tested in WB, ICC/F, IHC-P, IHC-Fr, FACS, IP, and ELISA (https://www.genetex.com/Product/Detail/SARS-CoV-2-COVID-19-Nucleocapsid-antibody/GTX135357).

Eukaryotic cell lines

Policy information about cell lines and Sex and Gender in Research

Cell line source(s)	HEK-293T cells (ATCC CRL-3216) and the derivative expressing hACE2, ie 293T/hACE2.cl22 (generated at Rockefeller University, J Exp Med (2020) 217 (11): e20201181.<br>https://doi.org/10.1084/jem.20201181); Caco-2 cells (ATCC HTB-37™); human hepatoma-derived Huh-7.5 cells (generated at Rockefeller University https://doi.org/10.1128/JVI.76.24.13001-13014.2002<br>); Vero E6 cells and a derivative expressing TMPRSS249 (CRL-1586) ; A549 cells (CRM-CCL-185)
Authentication	Not authenticated after purchase from ATCC.
Mycoplasma contamination	All cell lines tested negative for mycoplasma contamination.
Commonly misidentified lines<br>(See ICLAC register)	no commonly misidentified cell lines were used in the study

Animals and other research organisms

Policy information about studies involving animals; ARRIVE guidelines recommended for reporting animal research, and Sex and Gender in Research

Laboratory animals	AlivaMab Mouse (Ablexis LLC); six-week old hACE2-knock-in female mice, in which human ACE2 cDNA replaces the endogenous mouse ACE2 sequences, were obtained from Jackson Labs (B6.129S2(Cg)-Ace2tm1(ACE2)Dwnt/J, strain 035000).
Wild animals	no wild animals were used in the study
Reporting on sex	No sex- and gender-based analysis was done since it is irrelevant to this study. We followed the procedure used in the Nature paper (Zhou B, 2021, Nature doi: 10.1038/s41586-021-03361-1), including animal sex.
Field-collected samples	no field collected samples were used in the study.
Ethics oversight	the Rockefeller University Institutional Animal Care and Use Committee (IACUC)

Note that full information on the approval of the study protocol must also be provided in the manuscript.

Flow Cytometry

Plots

Confirm that:

☒ The axis labels state the marker and fluorochrome used (e.g. CD4-FITC).

☒ The axis scales are clearly visible. Include numbers along axes only for bottom left plot of group (a 'group' is an analysis of identical markers).

☒ All plots are contour plots with outliers or pseudocolor plots.

☒ A numerical value for number of cells or percentage (with statistics) is provided.

Methodology

Sample preparation	The ACE2 expressing cells were detached from plates with 10 mM EDTA in PBS and then incubated in the absence or the presence of human anti-hACE2 mAbs (2 μg/ml) for 2 hr at 4°C. After washing, the cells were incubated with AlexaFluorTM 488 (or Alexa FluoTM 647 when indicated) conjugated goat anti-human IgG (ThermoFisher Scientific).
Instrument	Attune® NxT Acoustic Focusing Cytometer (ThermoFisher Scientific)

Software	AttuneTM NxT software v3.1.2 &v5.1.0
Cell population abundance	100,000 cells were used per sample.
Gating strategy	First we used FSC/SSC to gate the "cells", followed by FSC-H and FSC-A to gate the "Single cells".

☒ Tick this box to confirm that a figure exemplifying the gating strategy is provided in the Supplementary Information.
